# Tumor-selective dye-based histological electrophoresis enables intraoperative tumor diagnosis via tumor-specific enhancement

**DOI:** 10.7150/thno.105500

**Published:** 2025-01-06

**Authors:** Feiran Zhang, Jianing Cheng, Xu Peng, Chengbin Zhang, Limei Qu, Songling Zhang, Junhu Zhang, Shoujun Zhu

**Affiliations:** 1 Joint Laboratory of Opto-Functional Theranostics in Medicine and Chemistry, The First Hospital of Jilin University, Changchun 130021, P. R. China; 2 State Key Laboratory of Supramolecular Structure and Materials, Center for Supramolecular Chemical Biology, College of Chemistry, Jilin University, Changchun 130012, P. R. China; 3 Department of Hepatobiliary and Pancreatic Surgery, The First Hospital of Jilin University, Changchun 130021, P. R. China; 4 Department of Pathology, The First Hospital of Jilin University, Changchun 130021, P. R. China.; 5 Department of Obstetrics and Gynecology, The First Hospital of Jilin University, Changchun 130021, P. R. China.

**Keywords:** Three-dimensional histological electrophoresis, Cancer diagnosis, Tumor-seeking dye

## Abstract

Solid tissue biopsy is fundamental in guiding surgeons during intraoperative and peri-operative management of cancer patients. However, conventional histopathologic methods depend heavily on the expertise of trained pathologists, facing challenges in accuracy and efficiency.

**Methods:** Here, we show that unbiased labeling of proteins within tissue sections using tumor-selective dyes enhances tumor-specific signals, enabling robust and accurate differentiation of tumors from normal tissues in less than 45 min. This diagnostic approach combines a tumor-selective dye labeling strategy and a three-dimensional (3D) histological electrophoresis separation strategy to visualize protein differences between tissues and exclude off-target interference.

**Results:** We successfully diagnose and delineate malignant tissue from frozen and fresh surgical specimens from 34 patients across six types of cancer (mean AUC = 0.93). Furthermore, we apply this method to distinguish different histological characteristics in liver cancer surgical specimens, as well as identify and quantify the degree of inflammation in tumor-surrounding tissues.

**Conclusion:** This rapid, accurate, unbiased, and marker-free approach may enhance intraoperative detection of multiple types of tumor specimens.

## Introduction

During tumor surgery, rapid and accurate histopathological diagnosis is essential for clinical decisions. However, the conventional method of intraoperative consultation pathology is intensive in time, labor, and costs, and relies heavily on the expertise of trained pathologists. Deep learning tools have achieved performance levels comparable to pathologists in certain diagnostic tasks 1-4. However, deep learning models are typically trained for specific diagnostic tasks, which means a single model cannot handle the complex clinical pathological work 5-8. This limitation makes them less competitive in intraoperative settings where the analysis of multiple types of cancer is required. Fluorescent probes have produced promising outcomes in enhancing the differentiation between tumors and normal tissues both *in vivo* and *ex vivo*. *In vivo*, fluorescent probes are used to guide surgical resection of solid tumors in real time 9-11. The US Food and Drug Administration (FDA) approved near-infrared (NIR) dye, indocyanine green (ICG), has been extensively evaluated in fluorescence-guided surgery (FGS) 12-21. Although ICG is not tumor-specific, it can still highlight tumors due to its enhanced permeability and retention effect in tumors, followed by tumor cell internalization 22,23. However, the variable tumor uptake and incomplete clearance of fluorescent dyes reduce the accuracy of distinguishing tumors from normal tissues. Fluorescent probes for FGS have been developed to target tumor-specific molecular expression 24-31, enzyme activity 32-34, and abnormal physiology of the tumor microenvironment (TME), including hypoxia 35, increased lysosomal viscosity 36-38, and acidic pH 39,40. Unfortunately, regardless of the targeting mechanism, tumor uptake of fluorescent probes can be highly variable, with considerable off-target accumulation in normal tissues and organs, resulting in poor sensitivity and high false-positive rates 14,41,42. More accurate tumor diagnosis can potentially be achieved by analyzing surgical tissues using fluorescence lifetime (FLT) imaging in FGS 43. However, the FLT strategy requires specialized equipment and costs, which may limit its widespread usability, particularly in smaller pathology centers.

In contrast to *in vivo* diagnosis, which depends on the uptake of fluorescent probes by tumor tissues, *ex vivo* diagnosis of the tissue sections from surgically resected tissue using fluorescent probes depends on the overall differences of biomarkers between malignant and normal tissues. Previous studies have identified notable differences in types and expression levels of protein between cancerous and healthy tissues 44-49. To address this, a series of tumor-selective dyes have been developed to identify the overall protein differences between malignant and normal tissues in an unbiased and marker-free manner 50. By designing the structure of chlorine-containing dyes, it is possible to modulate the covalent binding of meso-chlorine on the cyclohexyl ring of dyes to specific thiol groups in various tumor signature proteins, forming stable dye@protein complexes in situ, thereby leading to increased brightness 50. This approach helps avoid errors caused by a single marker and enables the visualization of tumor regions within tissue sections, with potential as an intraoperative diagnostic pipeline. Moreover, the tumor-specific shift in signal intensity can accurately distinguish tumors from normal tissues, achieving a 100% accuracy rate in a small cohort of breast and lung cancer 50.

**Figure 1:**
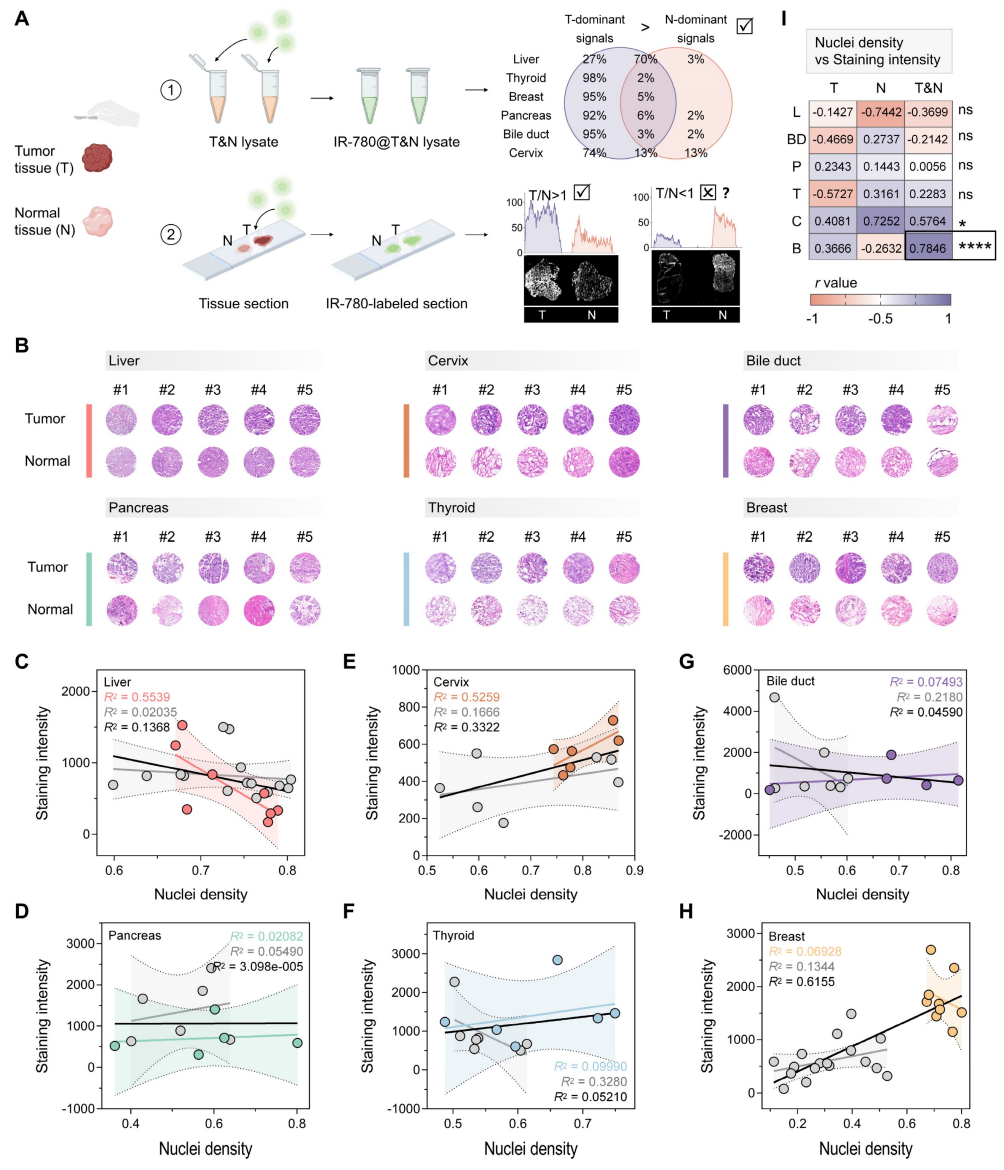
** Protocol for evaluating the tumor identification effect of tumor-selective dyes.** (A) Schematic workflow illustrating the concept of distinguishing tumors from surgical specimens via tumor-selective dye (IR-780) labeled tissue lysate and tissue sections, created with BioRender.com. For six types of prevalent tumor (liver, thyroid, breast, pancreas, bile duct, and cervix), the signals of IR-780@proteins in tumor tissue lysate are notably higher than in normal tissue lysate, enabling reliable tumor identification. However, when IR-780 is applied to tissue sections, the signals quantified from tumor regions are not consistently greater than that quantified from normal tissue regions, suggesting the influence of off-target signals in the tissue section staining protocol. (B) H&E analysis of nuclei density in the six types of tumor and their corresponding normal tissues (liver, n = 7; cervix, n = 6; bile duct, n = 5; pancreas, n = 5; thyroid, n = 6; breast, n = 9). (C-H) Correlation of the staining intensity of IR-780-labeled tissue sections and the nuclei density of tissue sections for liver (C), pancreas (D), cervix (E), thyroid (F), bile duct (G), and breast (H). Trendlines are shown for tumor (color-coded), normal tissue (gray), and combined tumor and normal tissue samples (black). The *R^2^* values represent the coefficient of determination, reflecting the goodness of fit. Each data point corresponds to one tissue. (I) Pearson correlation (*r*) analysis predicts the effectiveness of the direct staining protocol in tissue sections of different tumor types. In breast cancer (*r* = 0.7846, ****P < 0.0001), tumor regions can be properly diagnosed based on staining intensity [liver (L), thyroid (T), breast (B), pancreas (P), bile duct (BD), and cervix (C)].

When performing unbiased labeling analysis of tissue section proteins using tumor-selective dyes, an important question is whether the detected fluorescent signal faithfully represents the distribution of malignant tissue in tissue sections. In practice, fluorescence intensity quantification directly on tumor-selective dye-stained sections may be interfered by the unbound free dyes, which reduces the reliability of distinguishing tumors from normal tissues on signal intensity. Some free-dye molecules may adsorb to tissue sections and are affected by tissue characteristics such as tissue cell type, nuclei density, and permeability. In addition, certain tumor-selective dyes can insert into protein structures via supramolecular interactions, forming non-covalent binding, which can result in off-target fluorescence. As shown in **Figure 1 and S1**, for cancers such as liver cancer, cholangiocarcinoma, and pancreatic cancer, substantial accumulation of non-tumor-specific fluorescence masks the dye@protein complex signal of interest, making it difficult to quantify tumor-specific contrast at an absolute scale across patients, which severely limits the clinical benefit of tumor-selective dyes (**Figure 1 and S1**). Therefore, there is a pressing need for a rapid and accurate method to spatially identify covalent signals (tumor-selective dye@protein) within tissue sections and facilitate intraoperative tumor diagnostics.

3D histological electrophoresis is a method to adequately remove dyes that are non-covalently bound to the molecular structure of proteins, allowing us to quickly read and analyze the covalent signal of interest with histological information (**Figure S2**). As shown in **Figure S2B**, after power-up, the flow order of anions in the electrophoresis device is as follows: negative electrode, electrophoresis buffer, stacking gel, separating gel, electrophoresis buffer, and finally the positive electrode. The molecules to be separated move from the negative electrode to the positive electrode. Under the influence of a parallel electric field, free dyes are efficiently separated from the proteins in tissue sections, allowing us to identify the tumor-selective dye-labeled proteins without interference from free dyes.

Combined with **Figure S3** and our previous research 51,52, 3D histological electrophoresis strategy can distinguish tumors from normal tissues with a high tumor-to-normal ratio in breast cancer and cervical cancer. In this work, we present clinical studies on a broader range of tumor types based on tumor-selective dye labeling and histological electrophoresis separation strategies, particularly focusing on the tumor types with high off-target dye accumulation in normal tissues, such as liver cancer, cholangiocarcinoma, pancreatic cancer, and esophageal carcinoma. We examined clinically collected frozen and fresh biopsy samples of the above tumor types and found that after histological electrophoresis separation, there was a considerable shift in signal intensity in different regions within the tissue section, which was consistent across tumor types in multiple patients. The post-electrophoresis signal intensity faithfully represents the tumor-selective dyes covalently bound to proteins, which can increase the tumor-to-normal tissue ratios by several folds. Furthermore, our findings indicate that the Tumor-Selective Dye-based Histological Electrophoresis (TSD-HE) strategy can accurately assess the inflammatory and malignant status of tumor tissues. Our procedure can sensitively detect pathological changes in biopsies within 45 min, making it a promising complementary tool for clinical intraoperative tissue diagnosis.

## Results and Discussion

### Analysis of tumor-selective dye@protein signals and off-target signals in different tumor types

We first analyzed the differences in tumor-selective dye@protein signals between six tumors and their coexisting normal tissues. After screening substituents (H, Cl, and Br) on the cyclohexyl ring, as well as evaluating side chain length and functional groups, the dye IR-780, exhibiting optimal tumor selectivity, was selected for further investigation 50. We extracted proteins from the tumors and corresponding normal tissues of the liver, thyroid, breast, pancreas, bile duct and gallbladder, and cervix (three patients for each tumor type, **Figure S4A**). Next, we labeled the above lysates with IR-780 to obtain six IR-780@tumor lysates and six IR-780@normal tissue lysates. After electrophoresis separation, we quantified the signals of IR-780@tumor lysate and IR-780@normal tissue lysate across the whole molecular mass range, and calculated the ratio of tumor-to-normal tissue signal at each molecular mass. To increase the robustness, we set thresholds of 1.25 and 0.80 for ratio analysis. A tumor-to-normal tissue ratio exceeding 1.25 indicates a tumor-dominant signal, defined as a protein signal that aids in tumor identification. Conversely, a ratio below 0.80 is defined as a normal tissue-dominant signal. After IR-780 labeling, tumor-dominant signals of the six types of tumor accounted for a larger proportion of the entire molecular mass range than that of normal tissues (**Figure 1A and S4B-G**). For the surgical specimens from thyroid, breast, pancreas, and bile duct and gallbladder, the tumor-dominant signals accounted for more than 90% of the entire molecular mass range. In surgical specimens of cervical cancer, the tumor-dominant signals accounted for 74% of the entire molecular mass range, while the normal tissue-dominant signals were only 13%. The tumor-dominant signals analyzed in the surgical specimens of liver cancer were 27%, which were higher than that of normal tissue (3%). This analysis demonstrates that IR-780 labeling significantly enhances tumor-specific signals, which can be used to distinguish tumors from normal tissues with high accuracy.

However, in pilot experiments, we found that the signals from tumor regions were not always higher than those of normal tissue regions after direct IR-780 staining of tissue sections (**Figure 1A and S1A**). The post-staining signals were tissue-dependent. We hypothesized that the intensity of non-specific signal was primarily influenced by parenchymal cell density, while the intensity of specific signal was dominated by the protein content that can be labeled. Therefore, whether a certain type of tumor could be distinguished by the direct staining approach depended largely on whether the non-specific signal was synergistic or antagonistic with the specific signal. We conducted a detailed analysis of the nuclei density and post-staining signal intensity of the above tumors and their corresponding normal tissues (**Figure 1B-H**). In breast cancer, the nuclei density in tumor regions was significantly higher than that in normal tissue regions (*P* < 0.0001, **Figure S1B**), resulting in higher non-specific adsorption in tumor regions compared to normal tissue (**Figure S1A**). There was a synergistic relationship between non-specific and specific signals, which jointly enhanced the highlighting of malignant regions (*R*^2^ = 0.6155 for breast cancer, **Figure 1H**; **Figure 1I and S1C**). In contrast, there was no significant difference in nuclei density between tumors and normal tissues of the liver, pancreas, cervix, and thyroid (**Figure S1B**), and an overall antagonistic relationship between non-specific and specific signals was noted (*R*^2^ = 0.1368 for liver, **Figure 1C**; *R*^2^ = 3.098e-005 for pancreas, **Figure 1D**; *R*^2^ = 0.3322 for cervix; *R*^2^ = 0.05210 for thyroid, **Figure 1F**; **Figure 1I**). This antagonism resulted in instances where signals were sometimes higher in tumor regions and other times higher in normal tissue regions (**Figure 1A and S1A, D**).

Notably, although there was a significant difference in nuclei density between tumor and normal tissue regions in cholangiocarcinoma specimens (*P* = 0.0348, **Figure S1B**), the malignant regions were not fully identifiable based on the staining results (**Figure S1A**). This outcome could be attributed to the insufficient synergy between non-specific and specific signals in cholangiocarcinoma surgical specimens (*R*^2^ = 0.04590, **Figure 1G**; **Figure 1I**). Therefore, the difference in nuclei density between the tumor and adjacent normal tissue (with a recommended threshold of *P* < 0.0001) could serve as a predictive marker of whether a certain type of tumor can be diagnosed by direct staining. More importantly, the above results indicated that effectively minimizing non-specific adsorption is critical for expanding the clinical applicability of tumor-selective dyes.

### Tumor-selective dye@proteins identified by 3D histological electrophoresis distinguishes tumors from normal tissues

**Figure 2:**
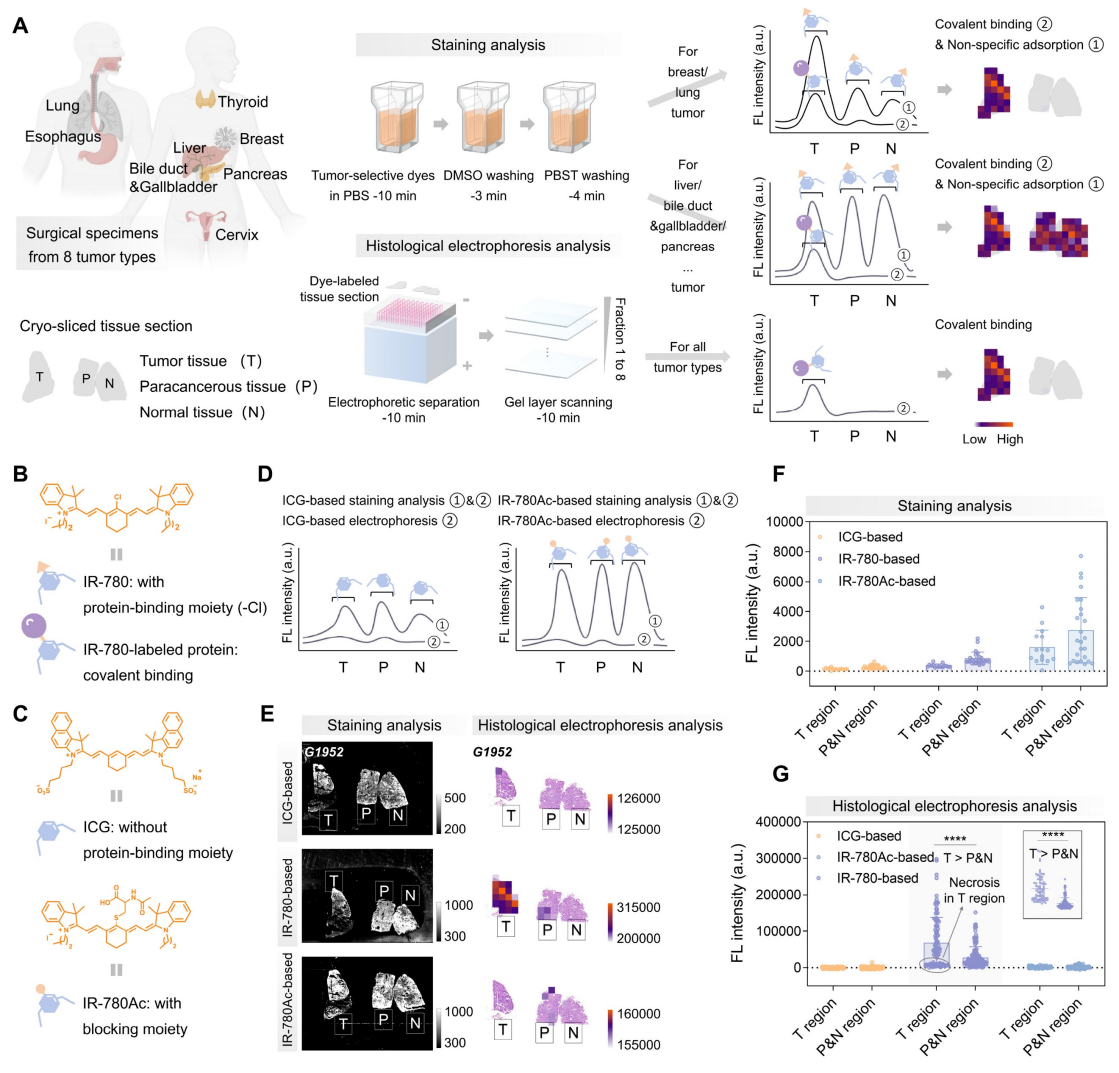
** Purification of non-specific signals after tumor-selective dye staining by histological electrophoresis.** (A) Schematic representation of the identification of histological types by covalent binding signals within tissue sections. Comparison of signals collected after IR-780-based staining analysis and IR-780-based histological electrophoresis analysis. The signals after staining analysis are derived from dyes that covalently bind to proteins within tissue sections and dyes that are non-specifically adsorbed to tissue sections. The signals after histological electrophoresis analysis are derived from dyes that covalently bind to proteins within tissue sections. (B) Chemical structure of IR-780 and schematic representations of IR-780 and IR-780-labeled protein. (C) Chemical structures and schematic representations of ICG and IR-780Ac. The binding behavior of ICG and IR-780Ac to proteins is different from that of IR-780 to proteins. Due to the absence of binding sites, ICG and IR-780Ac are unable to form stable covalent bonds with proteins. (D-G) Comparison of ICG-/IR-780-/IR-780Ac-based staining analysis and ICG-/IR-780-/IR-780Ac-based histological electrophoresis analysis in the liver cancer specimens from 3 patients (G1952, G0211, and S0002, **Table S1**). (D) Comparison of signals collected after staining analysis and histological electrophoresis analysis based on ICG and IR-780Ac. (E) Fluorescence intensity image describes staining analysis results. Heat map describes histological electrophoresis analysis results. The co-localization of H&E staining results and histological electrophoresis analysis results of tissue sections to describe the spatial distribution and abundance of IR-780 covalently bound proteins in tissue sections. Heat map represents the total signal of protein fractions after histological electrophoresis separation (fractions 1 to 8). (F and G) Plots of the signal intensity of multiple ROIs in tumor region and paracancerous and normal region after ICG-/IR-780-/IR-780Ac-based staining analysis (The number of ROIs is five, and the size of ROIs is 1 mm × 1 mm, F) and ICG-/IR-780-/IR-780Ac-based histological electrophoresis analysis (ROIs: n > 10, G). Top right of (G): Plot of the signal intensity of ROIs in tumor region and paracancerous and normal region after IR-780-based histological electrophoresis analysis after excluding signals of the necrotic region from G0211. Significant differences in signal intensity are observed between the tumor region and paracancerous and normal region (****P < 0.0001).

3D histological electrophoresis has the efficacy in removing large amounts of non-tumor-specific fluorescence accumulated in tissue sections, making it a good tool for tissue diagnosis based on tumor-selective dyes (**Figure 2A**). We further designed experiments to ensure that the signals quantified from post-electrophoresis gel layers (fractions 1 to 8) arises specifically from dye-labeled proteins. Following the interaction mechanism between tumor-selective dyes and proteins, IR-780 can insert into proteins through supramolecular interactions, followed by covalently binding by nucleophilic substitution reaction between the protein's —SH group and the Cl—C bond in Cl-containing dyes 53-58 (**Figure 2B**). To confirm that post-electrophoresis signals arose from covalent binding rather than non-specific accumulation, we designed and synthesized the IR-780Ac molecule by blocking the reaction site (-Cl) of IR-780 (**Figure 2C and S5**), ensuring that IR-780Ac could not form covalent bonds with protein molecules 59. We also analyzed ICG (**Figure 2C and S5**), which accumulates in tumors *in vivo* but lacked *ex vivo* tumor selectivity 22. We expected that ICG and IR-780Ac would interact non-covalently with proteins within tissue sections during staining analysis and could be completely removed by subsequent electrophoresis separation (**Figure 2D**). In contrast, the covalent signals of interest were fully revealed after the histological electrophoresis analysis of IR-780-stained tissue sections, representing the tumor selectivity of IR-780.

We labeled a mixture of protein standards—bovine serum albumin (BSA, 66.4 kDa) and β-lactoglobulin (β-LG, 18.4 kDa)—with ICG, IR-780, and IR-780Ac, respectively. As expected, ICG and IR-780Ac were unable to form covalent bonds with the protein molecules, and were completely separated from protein fractions under the electric field (**Figure S2C**). In contrast, IR-780@BSA and IR-780@β-LG were identified as fractions 1 and 2 at their corresponding molecular mass locations, demonstrating that the post-electrophoresis fraction signals could be fully attributed to the dye covalently bound to proteins (**Figure S2C**, center).

The next aim was to in situ identify tumor-selective dye@protein within tissue sections and eliminate off-target signals by 3D histological electrophoresis. We selected liver cancer surgical specimens as an ideal model to validate the effectiveness of 3D histological electrophoresis in removing off-target signals and identifying covalent signals. Liver cancer specimens were particularly suitable for this validation because, compared to other tumor types, they are characterized by lower tumor-dominant signals (27%), higher normal tissue-dominant signals (3%), and a higher proportion (approximately 80%) and denser arrangement of parenchymal cells. This combination results in strong non-specific dye accumulation sufficient to interfere with the directly staining diagnosis. Moreover, the comparison between tumor-dominant signals (27%) and normal tissue-dominant signals (3%) suggests that proteins selectively labeled by IR-780 in liver tumors could serve as targets for tumor identification via 3D histological electrophoresis.

**Figure 3:**
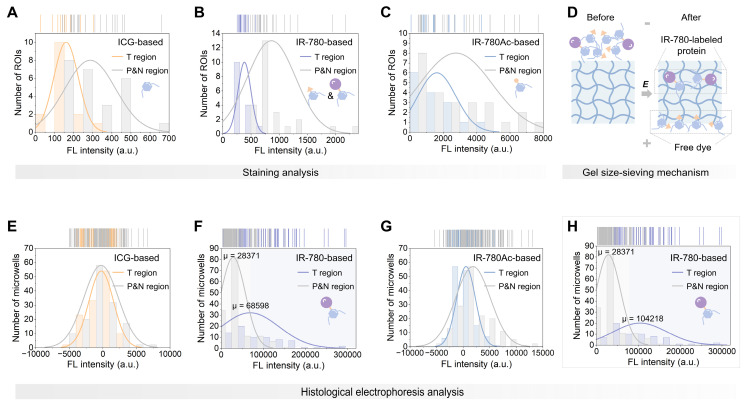
** Illustrative plots of covalent binding signals in different regions within liver cancer tissue sections.** (A-C) Histograms of signals after ICG-based (A), IR-780-based (B), and IR-780Ac-based (C) staining analysis in tumor region and paracancerous and normal region from the same tissue section of liver cancer specimen. (D) Schematic representation of the gel size-sieving mechanism for free dye removal. (E-H) Histograms of signals after ICG-based (E), IR-780-based (F), and IR-780Ac-based (G) histological electrophoresis analysis in tumor region and paracancerous and normal region from the same tissue section of liver cancer specimen. (H) Histogram reflects the signal distribution after IR-780-based histological electrophoresis analysis after excluding the necrotic region signals (from G0211).

We collected specimens (G1952, G0211, and S0002) from liver cancer patients for the following investigations. Firstly, we evaluated tissue sections from the above patients using ICG, IR-780, and IR-780Ac-based staining analysis, and quantified the post-staining signals from different regions (tumor, paracancerous, and normal tissue regions) within tissue sections. Our results showed that the post-staining signal intensity in the tumor regions was considerably overlapped with that of the paracancerous and normal regions (**Figure 2E-F and 3A-C**). Since the normal liver could accumulate a large amount of off-target signal, the post-staining signal intensity in the tumor regions was even lower than that of the paracancerous and normal regions.

Next, we performed electrophoresis analysis on the ICG, IR-780, and IR-780Ac-stained tissue sections. We observed that, as expected by the size-sieving mechanism (**Figure 3D**), no valid signals that were contrastable from the background signals were detected in the gel layers corresponding to all fractions after histological electrophoresis analysis based on ICG and IR-780Ac (G1952 in **Figure S6**, S0002 in **Figure S7**, and G0211 in **Figure S8A-C**). This demonstrated the effectiveness of the 3D histological electrophoresis method in removing non-specific interferences and identifying the covalent signal of interest. The signals of the eight fractions were summed as the final result of histological electrophoresis analysis to maximize the signal differences between tumor and normal tissues. By quantifying signals from the different tissue regions, we demonstrated that covalent signals from the tumor regions within tissue sections were significantly higher than those quantified from the paracancerous and normal regions (IR-780-based group, *P* < 0.0001, **Figure 2G**). We performed a normal distribution analysis of the signals from different regions after histological electrophoresis analysis (**Figure 3E-G**). The signals after IR-780-based histological electrophoresis analysis yielded a ∆µ of 40227 between the tumor regions and the paracancerous and normal regions (**Figure 3F**), showing excellent tissue contrast of covalent signals.

In addition, we noted that the tissue sections containing necrotic cores (from G0211) did not show enhanced tumor-specific signal in their tumor regions after electrophoresis (**Figure 2G and S8**). This is due to the fact that protein expression in these non-viable tumor areas is extremely low compared to viable tumor cells 60. This observation was also supported by the analysis of tissue lysates (**Figure S9A**). The total signal intensity of proteins labeled with IR-780 in viable tumor lysate was higher than that in necrotic tumor lysate (**Figure S9B**). However, we do not anticipate that the lack of signal enhancement in necrotic region within tumor interior will not adversely affect the performance of TSD-HE in intraoperative tissue diagnosis since the boundaries of tumors are most relevant for clinical assessments and surgical resections 61. The poor enhancement in necrotic region instead illustrates that the quantified signals after histological electrophoresis analysis are contributed by the tumor signature proteins labeled by IR-780, demonstrating the ability of 3D histological electrophoresis to recognize covalent signals of interest and thus identify tumors. After removing the signals quantified from the necrosis (ensuring that the signals of tumor regions were solely from cancerous tissue), the ∆µ of the signals after TSD-HE analysis achieved 75847 between the tumor regions and the paracancerous and normal regions (**Figure 3H**). Notably, the removal of signals from necrotic tissues did not change the signal distribution of other groups (ICG and IR-780Ac groups, **Figure S10A-E**). The tumor-selective dye labeling strategy combined with the 3D histological electrophoresis separation strategy works synergistically to enhance tumor contrast (**Figure S10F**). These results confirm that TSD-HE serves as an ideal general analysis tool, setting up opportunities for highlighting tumors via tumor-selective dye@proteins.

### TSD-HE for assessing histological characteristics in liver cancer

**Figure 4:**
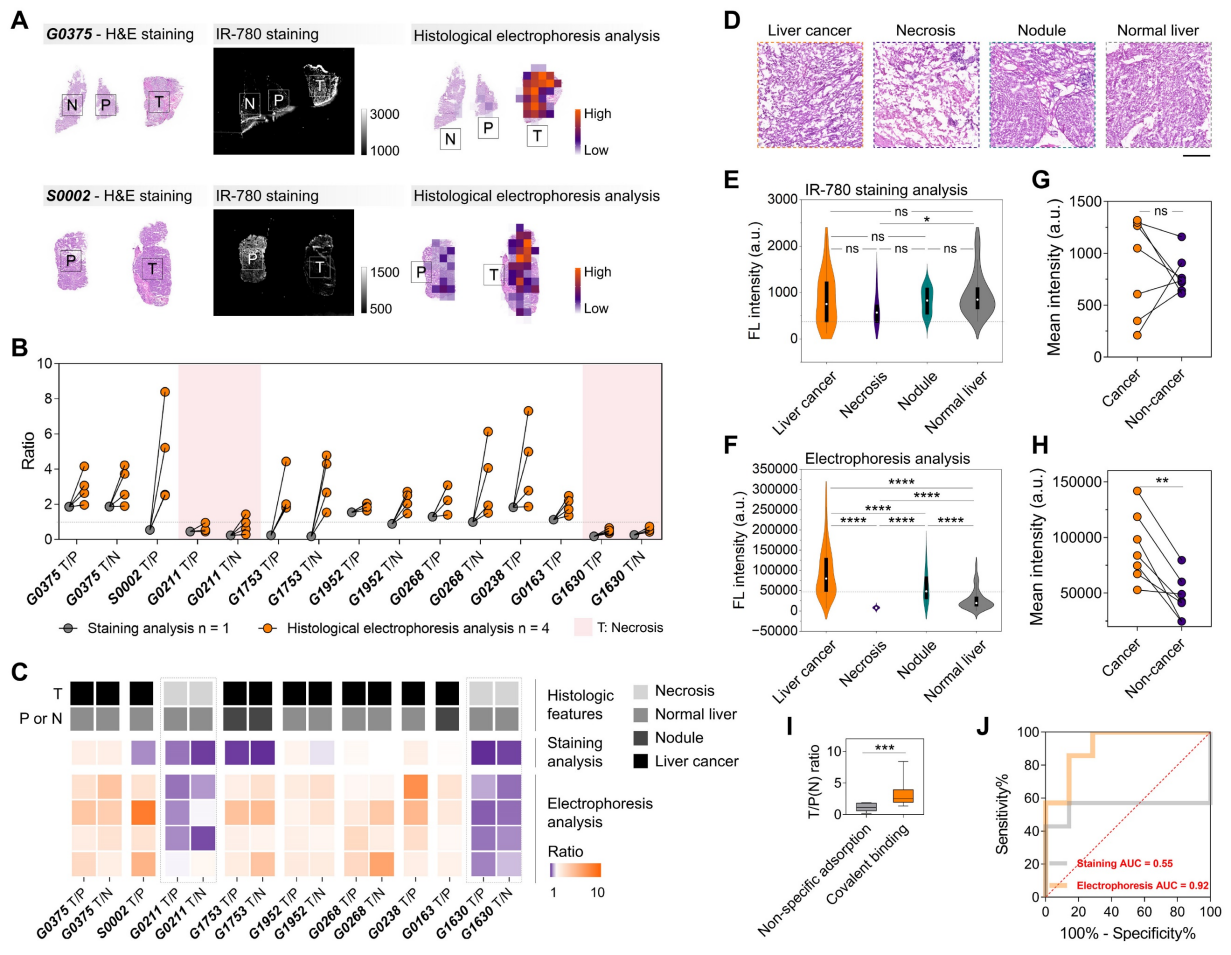
** Signals of IR-780-labeled proteins reflect histological characteristics in liver cancer.** (A) Compared to IR-780-based staining analysis, IR-780-based histological electrophoresis analysis faithfully represents the distribution of histological types in tissue sections. (B) Enhancement of tumor-to-paracancerous (or normal) tissue ratios in liver cancer via histological electrophoresis analysis. The specimens of liver cancer are obtained from nine patients (**Table S1**). (C) Association between tumor-to-paracancerous (or normal) tissue ratios and the histological types of tumor and paracancerous (or normal) tissue. (D) H&E staining results reflect four representative histological types (liver cancer, necrosis, liver nodule, and normal liver) in the surgically resected tissue of liver cancer. Scale bar: 500 µm. (E and F) Violin plots show the signal distribution after staining analysis (E) and histological electrophoresis analysis (F) in liver cancer and several normal tissue types across multiple patients (n = 9). Statistical significance is calculated using a *t*-test: *P = 0.0290 and ****P < 0.0001. (G and H) The patient-wise (n = 9) mean signal intensity of cancer (orange) and non-cancer tissue (purple), calculated as the mean of multiple ROIs (For staining analysis, the number of ROIs is five, and the size of ROIs is 1 mm × 1 mm, G; for histological electrophoresis, n > 10, H) of histologically identified cancer and non-cancer tissue. Statistical significance is calculated using a *t*-test: **P = 0.0068. (I) Comparison of the ratios calculated from the IR-780-staining strategy and IR-780-based electrophoresis separating strategy. Significant differences are observed between the covalent binding group and the non-specific adsorption group. (***P = 0.0004). (J) ROC plot of sensitivity% versus 100%-specificity% for cancer versus non-cancer classification in data from specimens across the above patients. The AUC is 0.92 for histological electrophoresis analysis versus 0.55 for staining analysis.

We further analyzed the frozen and fresh specimens from patients undergoing surgery for malignant liver tumors using IR-780-based histological electrophoresis (four tissue sections for each patient, **Table S1**), and compared them with the direct IR-780-staining tissue sections (one tissue section for each patient) to illustrate the tumor-specific enhancement with the removal of off-target signals. Under the influence of off-target accumulation, the quantified signals from tumor regions (direct IR-780-staining) were not always higher than those from paracancerous and normal regions, leading to unstable tumor identification by IR-780 staining (**Figure 4A-B**). Conversely, the post-electrophoresis signals (the summed heatmaps from the signals from fractions 1 to 8) in tumor regions were higher than those in paracancerous and normal regions, with a quantified mean tumor-to-paracancerous (or normal) tissue ratio of 2.975 (**Figure 4B**). Given the known challenges in the intraoperative application of tumor-selective dyes, a distinguishing aspect of this study is the direct one-to-one comparison of TSD-HE results with direct staining results and histology, along with the quantitation of key performance metrics including sensitivity and specificity. The hematoxylin&eosin (H&E) staining results of all tissue sections from collected surgical specimens were evaluated by two experienced pathologists into four histological groups: liver cancer, necrosis, nodule, and normal liver (**Figure 4C-D and S11**). The results showed that the IR-780 staining method could not usually distinguish tumors when liver nodules appeared in the surrounding tissue, suggesting that the non-specific adsorption of dye molecules by liver nodule tissues may be more obvious than in tumor tissues (**Figures 4B-C**). This phenomenon can be attributed to the fibrotic tissue inside the liver nodules. Notably, our data showed that TSD-HE could effectively distinguish tumors regardless of whether the surrounding tissue was nodular or normal liver parenchyma (**Figure 4B-C**). As expected, the post-electrophoresis signal corresponding to necrotic tumor tissue (G0211 and G1630) was lower than that of normal liver tissue (**Figure 4B**: pink area, **Figure 4C**: gray dashed line, and **Figure S11C, I**).

We next performed a global analysis of the post-staining and the post-electrophoresis signals of all specimens from nine patients and represented the data as violin plots across various identified histological types (**Figure S12**). The results showed a considerable overlap of the post-staining signals between the histological groups (**Figure 4E and S13A**). In contrast, the post-electrophoresis signals corresponding to liver cancer were significantly higher than those of all other histological types (*P* < 0.0001 for all groups, **Figure 4F**). In addition, we noted that the post-electrophoresis signal could significantly distinguish between liver nodules and normal liver parenchyma (*P* < 0.0001, **Figure 4F**; ∆µ = 29569, **Figure S13B**). Intraoperative evaluation of the tissues surrounding liver tumor is becoming increasingly important, as the level of inflammation is associated with the risk of disease recurrence. Thresholds extracted from the distribution analysis of post-electrophoresis signals were able to distinguish histological characteristics in liver cancer tissue sections, thereby enhancing the visual assessment based on histomorphology (**Figure S13C**). This indicates that the TSD-HE method could not only classify tumor versus normal tissue in liver cancer surgical specimens, but also provide more detailed histological characterization.

A patient-wise analysis (when paired cancer and non-cancer regions were available, n = 7) showed that the mean post-staining signal intensity varied widely between patients, with three patients showing higher signal intensity in the non-cancer tissues compared with the cancer tissues (**Figure 4G**). However, all the patient-wise mean post-electrophoresis signals in cancer regions were significantly higher than in non-cancer regions (*P* = 0.0068, **Figure 4H**). Compared with post-staining signals, the post-electrophoresis covalent signals showed ideal inter-patient stability in identifying malignant tissue (**Figure 4I**). We sought to quantify the accuracy of post-staining signals and post-electrophoresis signals in detecting cancer and non-cancer regions. A patient-wise receiver operating characteristic (ROC) analysis for cancer/non-cancer classification (defined by histology) resulted in an accuracy (area under the curve, AUC) of 92% for post-electrophoresis covalent signals and an accuracy of 55% for post-staining signals (**Figure 4J**). Comparison of diagnostic results between staining analysis and histological electrophoresis analysis illustrates that histological electrophoresis is essential to identify malignant regions within tissue sections based on the difference in fluorescence signal of tumor-selective dye@proteins between malignant and benign tissues. Collectively, TSD-HE analysis of surgical specimens from liver cancer patients can stably and accurately distinguish between cancer cell infiltrated regions, inflammation and nodule regions, and normal liver regions. This method has the potential to evaluate malignant margins during surgery and suggest intraoperative and post-operative strategies.

### TSD-HE for surgical specimen diagnosis of esophageal carcinoma, cholangiocarcinoma, and pancreatic cancer

***Esophageal carcinoma.*** We collected fresh specimens from esophageal carcinoma patients (n = 3, **Table S1**) and performed intraoperative tissue diagnosis by direct staining and TSD-HE strategies (**Figure S14A**). After electrophoresis, which removed the accumulated off-target signals in the paracancerous tissues (epithelial and subepithelial mucosa and connective tissues), we could distinguish malignant regions from benign regions based on the post-electrophoresis covalent signals (**Figure S14B-D**).

**Figure 5:**
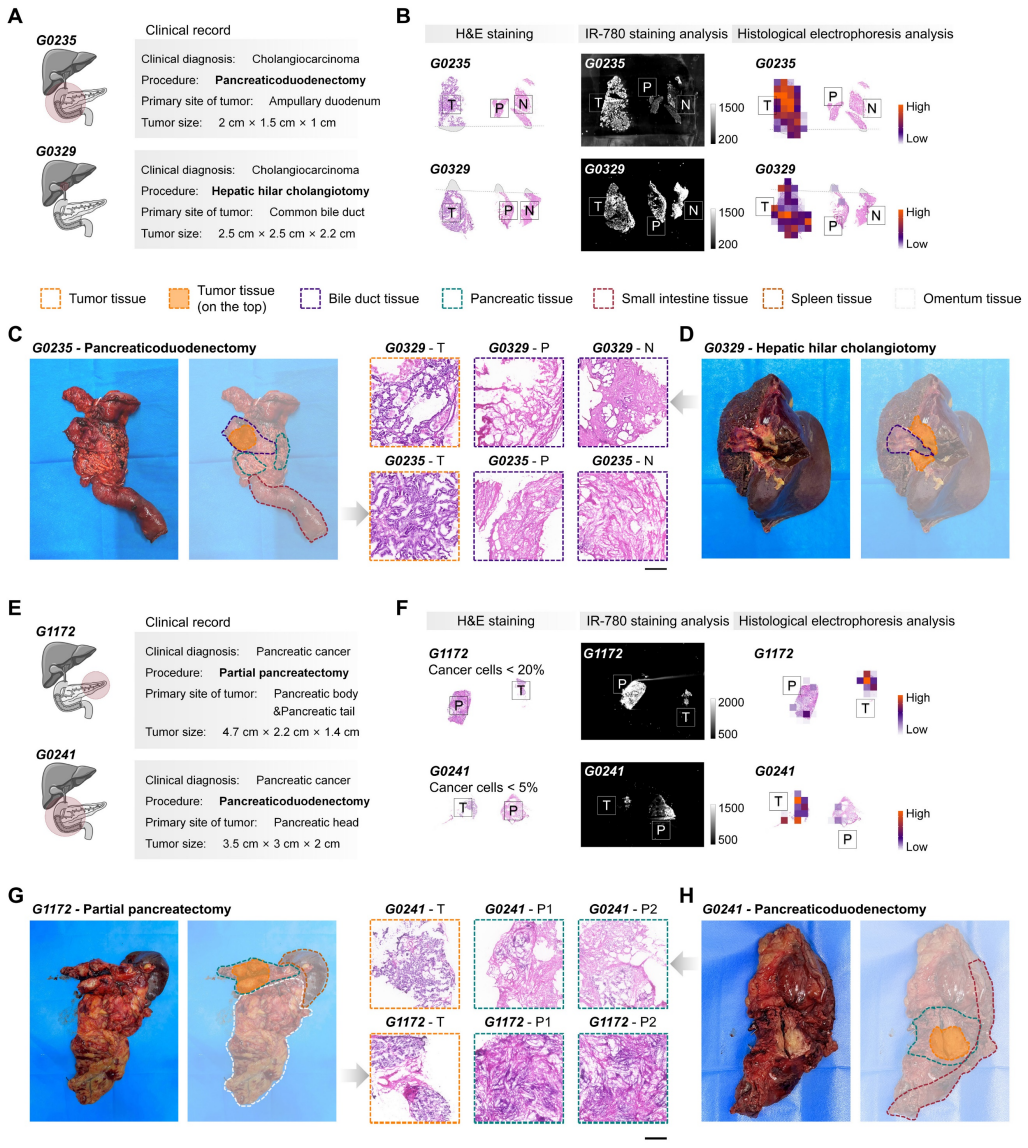
** Distinguishing tumors from normal tissues in cholangiocarcinoma and pancreatic cancer surgical specimens using TSD-HE.** (A) Cholangiocarcinoma at different primary locations and the corresponding clinical resection range (pancreaticoduodenectomy for G0235 and cholangiocarcinoma resection for G0329). (B) Comparison of IR-780-based staining analysis and IR-780-based histological analysis in the cholangiocarcinoma specimens from above patients. (C and D) Photographs and H&E staining results of flesh resected cholangiocarcinoma specimens obtained from G0235 (C) and G0329 (D). Scale bar: 500 µm. Dashed lines indicate the clinically identified tissue boundary. (E) Pancreatic cancer at different primary locations and the corresponding clinical resection range (partial pancreatectomy for G1172 and pancreaticoduodenectomy for G0241). (F) Comparison of IR-780-based staining analysis and IR-780-based histological analysis in the pancreatic cancer specimens from above patients. (G and H) Photographs and H&E staining results of flesh resected pancreatic cancer specimens are obtained from G1172 (G) and G0241 (H). Scale bar: 500 µm. Dashed lines indicate the clinically identified tissue boundary.

***Cholangiocarcinoma and pancreatic cancer.*** We next sought to examine our method by detecting tumors from surgical specimens of cholangiocarcinoma (n = 6, **Table S1**) and pancreatic cancer (n = 8, **Table S1**). The differences in the proteins labeled by tumor-selective dyes between the tumors of cholangiocarcinoma and pancreatic cancer, and the corresponding paracancerous and normal tissues, suggest that these tumors can be effectively identified by the post-electrophoresis covalent signals (**Figure 1A and S4C-D, G**). However, unlike simpler tumor types such as breast and thyroid cancer, where surgery specimens usually include only tumor and adjacent normal tissues, surgical specimens from tissues such as gallbladder, bile ducts, and pancreas may be influenced by the primary tumor location and the surgical procedure performed. To promote the use of TSD-HE as a routine intraoperative diagnostic tool, we evaluated its utility in different clinical situations in detail (**Figure 5**). We collected and analyzed intraoperative specimens for the pancreaticoduodenectomy (G0235, as a representative) and hepatic hilar cholangiotomy (G0329, as a representative), which are common procedures for cholangiocarcinoma tumors (**Figure 5A-D**). Unlike staining-based recognition, TSD-HE analysis was not affected by off-target signals. Our results demonstrated that TSD-HE could provide a rapid and unbiased prediction of malignant margins in surgical specimens, regardless of the primary locations of cholangiocarcinoma (**Figure 5B**).

For pancreatic cancer, partial pancreatectomy is usually performed when the tumor is located in the pancreatic body and tail (G1172, as a representative; **Figure 5E-H**). Conversely, tumors in the pancreatic head are typically treated with pancreaticoduodenectomy (G0241, as a representative; **Figure 5E-H**). Our results showed that the tumor-specific enhancement effect of TSD-HE is equivalent in surgical specimens from both primary tumor locations (**Figure 5F**). **Figure 5F** also illustrates the sensitivity of TSD-HE in detecting even small numbers of cancer cells, such as in tumors with low tumor cellularity and extensive desmoplastic tumor stroma, or in samples with nearly complete remission following chemo- or radiochemotherapy. A sample consisting of 95% normal and 5% cancerous cells (G0241) was accurately classified as malignant by TSD-HE. This aspect is especially important in clinical situations where intraoperative analysis is used to determine whether the operative margin is free of cancer, which can be particularly difficult when only a few cancerous cells are present.

**Figure 6:**
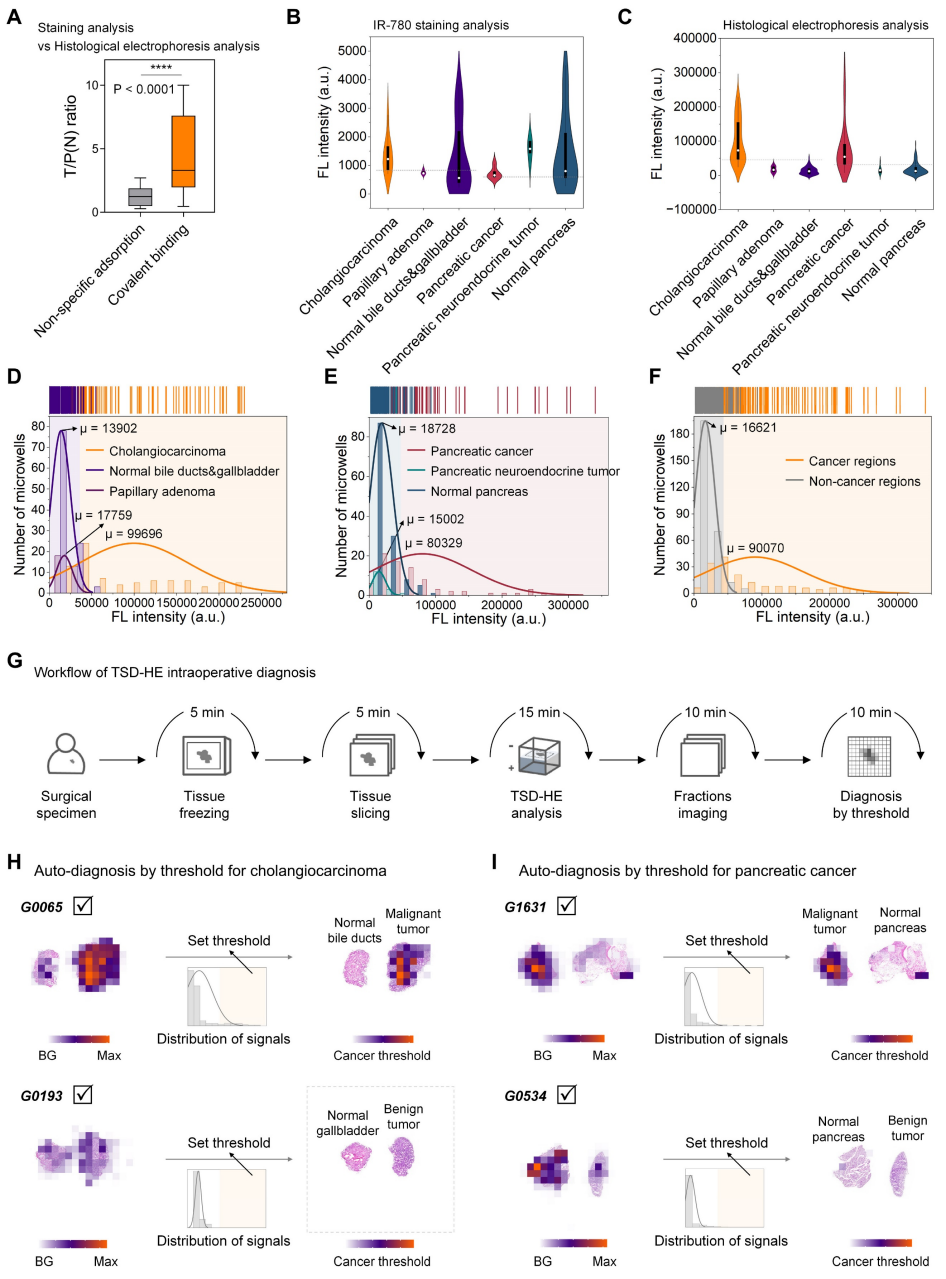
** Signals of IR-780-labeled proteins reflect histological characteristics in cholangiocarcinoma and pancreatic cancer.** (A) Comparison of the ratios calculated from IR-780-staining strategy and IR-780-based electrophoresis separating strategy. Significant differences are observed between the covalent binding group and the non-specific adsorption group. (****P < 0.0001). (B and C) Violin plots show the signal distribution after staining analysis (B) and histological electrophoresis analysis (C) in cholangiocarcinoma, pancreatic cancer, and several normal tissue types across multiple patients (n = 14). (D-F) Histograms of the covalent binding signals of different histological types within cholangiocarcinoma and pancreatic cancer tissue sections. (D) Histological types within the tissue sections from the above cholangiocarcinoma patients include: cholangiocarcinoma, normal bile duct and gallbladder, and papillary adenoma. (E) Histological types within the tissue sections from the above pancreatic cancer patients include: pancreatic cancer, pancreatic neuroendocrine tumor, and normal pancreas. (F) Signal histogram of cancer regions and non-cancer regions from the surgical specimens of cholangiocarcinoma and pancreatic cancer. (G) Schematic representation of the workflow of TSD-HE analysis for tissue sections of surgical specimens. (H) Automatically diagnosis of cancer region in the cholangiocarcinoma surgical specimens (from G0065 and G0193) via signal threshold. (I) Automatically diagnosis of cancer region in the pancreatic cancer surgical specimens (from G1631 and G0534) via signal threshold.

Next, we performed a comprehensive analysis of post-staining and post-electrophoresis signals from all specimens from 14 patients. The mean tumor-to-paracancerous (or normal) tissue ratio quantified in TSD-HE analysis (four tissue sections for each patient) was 4.709, which was significantly higher than that in direct staining analysis (*P* < 0.0001, one tissue section for each patient, **Figure 6A and S15**), despite the surgical specimens contained only a few cancer cells (for S0003, < 20%; for G0006, < 10%; **Figure S16**). A ROC analysis for cancer/non-cancer classification resulted in an accuracy (AUC) of 69% (in cholangiocarcinoma) and 68% (in pancreatic cancer) based on staining analysis (**Figure S15A, D**). In comparison, the accuracy, sensitivity, and specificity for cancer versus non-cancer classification based on TSD-HE were 98% (in cholangiocarcinoma) and 90% (in pancreatic cancer), respectively (**Figure S15C-D**).

We further histologically grouped our analyzed tissue sections into cholangiocarcinoma, papillary adenoma, normal bile ducts and gallbladder, pancreatic cancer, pancreatic neuroendocrine tumor, and normal pancreas, and represented the data as violin plots according to histological types. For staining analysis, the accumulated off-target signals in the stromal region exceeded the signals from cancer regions in both cholangiocarcinoma and pancreatic cancer, with no significant difference between histological groups (**Figure 6B**). In contrast, the post-electrophoresis covalent signals were highly tumor-specific, thereby clearly delineating cancerous tissues from surrounding tissues (**Figure 6C**). To quantify signal differences between histological types in cholangiocarcinoma and pancreatic cancer, we performed a normal distribution analysis of the above post-electrophoresis signals (**Figure 6D-E**). Our data demonstrated that the signal difference between cholangiocarcinoma malignancy and surrounding bile duct epithelium and stromal tissue was ∆µ of 85794, which is over six times than that of the normal tissues (µ value is 13902, **Figure 6D**). In addition, the intratumoral regions histologically defined as papillary adenoma within tumor could be well distinguished from malignant regions by covalent signal intensity (∆µ = 81937, **Figure 6D**), which is often challenging to identify in current pre-operative imaging and intraoperative rapid pathology. In pancreatic cancer, the ∆µ signal after TSD-HE analysis was 61601 between the benign and malignant tissue, and 65327 between the pancreatic neuroendocrine tumor (benign) and malignant tissue (**Figure 6E**).

As we showed in **Figure 5**, pancreaticoduodenectomy plays a role in the treatment of both cholangiocarcinoma and pancreatic cancer. During pancreaticoduodenectomy, guidelines require rapid pathological evaluation of both the common bile duct margin and the pancreatic margin of the surgical specimens 62. To address this need, we quantified the cross-tissue tumor enhancement ability of TSD-HE in cholangiocarcinoma and pancreatic cancer. Our data confirmed that TSD-HE could clearly delineate the common bile duct margins and pancreatic margins of the cancer cells, regardless of their primary tissues (∆µ = 73449, **Figure 6F**).

### Comparison of TSD-HE diagnosis and clinical pathological diagnosis

Pooled data from multiple patients suggest that, for a given tumor type, tumor and the co-existing normal tissue can be rapidly and automated distinguished during surgery using a post-electrophoresis covalent signal threshold (**Figure 6G**). Based on the threshold determined in **Figure 6F**, malignant regions within tissue sections could be visually identified without relying on specialized pathologists (G0065 as a representative for cholangiocarcinoma, **Figure 6H**; G1631 as a representative for pancreatic cancer, **Figure 6I**). Furthermore, we compared the diagnostic results from TSD-HE those reported by pathologists (based on H&E-stained images) in multiple cancer types, focusing on typical cancer regions and non-cancer regions (**Figure S17**). As shown in **Figure S17**, the mean quantified similarity between the two approaches exceeded 90%, demonstrating that TSD-HE could accurately, sensitively, and rapidly diagnose malignant regions within tissue sections.

Remarkably, diagnosis using this threshold did not confuse benign and malignant tumors, which is sometimes difficult to distinguish even with traditional morphological examination (G0193 as a representative, **Figure 6H and S18A-C**; G0534 as a representative, **Figures 6I and S18D**). While more precise thresholds would need to be iteratively refined through larger-scale clinical trials. Once established, the TSD-HE threshold could enable rapid, accurate, and automated determination of surgical margins and provide histological information, potentially addressing current challenges in the standardization and accuracy of image-based visual diagnosis.

## Conclusions

We have shown a quick, accurate, and simple method for diagnosing surgical specimens, suitable for multiple tumor types. This method involves selectively labeling proteins within tissue sections, followed by off-target signal removal by 3D histological electrophoresis. Within 45 min, proteins covalently labeled by Cl-containing NIR dye are imaged and quantified, and histological characteristics are identified from the heatmaps of quantified signals. This molecular diagnostic approach, based on protein molecules, is characterized by being unbiased and marker-free, which is advantageous when time is limited or when histological information is ambiguous. Our results show that the tumor-selective dye can label a variety of tumor signature proteins, and the resulting dye@protein can be used as multiple targets for rapid tumor identification. In addition, we have experimentally demonstrated that the 3D histological electrophoresis method can effectively remove non-specific signals accumulated in the tumor-surrounding tissues, addressing the application limitations of tumor-selective dyes in multiple types of tumor specimens. Specifically, we accurately and robustly distinguish tumors from normal tissues in breast cancer, cervical cancer, liver cancer, cholangiocarcinoma, pancreatic cancer, and esophageal carcinoma (four sections for each patient), indicating that this method can be used as an intraoperative specimen evaluation strategy with broad clinical application potential.

As shown in **Figure 1A and S4**, the differences between the tumor-dominant signals and normal tissue-dominant signals after IR-780 labeling vary across tumor types, which results in uneven discrimination in the diagnosis of different tumors. In breast cancer, thyroid cancer, pancreatic cancer, and cholangiocarcinoma, tumor-dominant signals are over 90%, enabling clear identification of tumor-positive regions within tissue sections at an ideal signal ratio of tumor-to-normal tissue. In liver cancer, however, the tumor-dominant signals are only 27%, although it is nine times higher than the normal tissue-dominant signals (3%), which can still distinguish tumor-positive regions. The development of dyes that are more selective for liver cancer-specific proteins will further improve the signal differences between liver cancer and surrounding normal tissues, allowing for diagnosis using a single signal threshold. In addition, future investigations involving larger patient cohorts will help establish the optimal signal threshold, which will further optimize the intraoperative pathology workflow and reduce the burden on pathologists.

Beyond the simple distinction between tumors and normal tissue, we also show that our method can rapidly assess the inflammation level in tissues surrounding liver tumors. Typically, the histologic assessment of inflammation level in the liver tissue around the tumor is performed in post-operative pathology (> 7 d) to indicate risk and guide treatment options. We show that the degree of tissue inflammation in liver specimens can be assessed intraoperatively through the signals of TSD-HE analysis. Furthermore, the signals from TSD-HE analysis can effectively distinguish mixed papillary adenomas from cancerous regions within tumors and exclude benign tumors by a single threshold. We envisage that this method can be utilized for rapid and automatic screening of biopsies that are confusing in imaging-based diagnosis, which is still lacking in clinical practice.

We anticipate that the large dataset generated from thousands of signals across multiple protein fractions and multi-dimensional information obtained by TSD-HE analysis is well suited for artificial intelligence (AI) approaches. In this study, we focused on benign and malignant tissue discrimination. By incorporating the data from TSD-HE analysis into machine learning models, we can gain a deeper understanding of the tissue protein signatures it represents. Future work will also focus on correlating signal differences with tumor malignancy scores, metastatic potential, and survival outcome after TSD-HE analysis to make up for the lack of understanding of the signals of tumor-selective-dye labeled proteins at this stage. Overall, our findings suggest that analyzing protein differences between benign and malignant tissues in surgical specimens using TDS-HE is a rapid and straightforward method for intraoperative tissue diagnosis. Notably, it can rapidly, accurately, and without reliance on markers or expertise, distinguish tumors from normal tissue in various types of cancer.

## Materials and Methods

The use of human samples in this study was approved by The First Hospital of Jilin University with informed parental consent, and the research was authorized by the ethics committee (K202119).

### Collection of clinical specimens and information

All human specimens (liver cancer, cholangiocarcinoma, pancreatic cancer, cervical cancer, breast cancer, and esophageal carcinoma) and clinical information analyzed in this study were obtained from The First Hospital of Jilin University. The collection process of liver cancer cases was described as an example. Post-operative specimens from liver cancer patients were stored at -80 ^o^C until analysis and intraoperative specimens of liver cancer were collected during surgery. The above surgical specimens were embedded into optimal cutting temperature (OCT) medium (Leica, 39475237) and cryo-sliced into 60-µm-thickness tissue sections for the immediate staining and histological electrophoresis analysis. The clinical information such as T stage, N stage, and patient age was also collected and analyzed from the pathological reports of these patients.

### Structural and purity characterization of dyes

^1^H-NMR spectra were obtained on Bruker AVANCE III 400 MHz NMR spectrometers (Q. One Instruments Ltd.). Coupling constants (J) are expressed in Hertz (Hz). Multiplicity was indicated as: s (singlet), d (doublet), t (triplet), and m (multiple). MS were recorded on QSTAR Elite (ABI). The operating conditions for high-resolution LC/MS were as follows: ESI^+^ spray voltage, 4.5 kV, or ESI-spray voltage, -3.5 kV; nebulizer gas, 1.5 L/min; drying gas, 100 kPa; heat block temperature, 200 ^o^C; CDL temperature, 200 ^o^C; IT Area Vacuum, 1.0×10^-2^ Pa; TOF Area Vacuum, 5×10^-4^ Pa.

### Structural and purity characterization of ICG

^1^H-NMR spectrum of ICG in DMSO. ^1^H NMR (400 MHz, DMSO-*d*_6_) δ 8.25 (d, *J* = 8.6 Hz, 2H), 8.09 - 8.01 (m, 4H), 7.97 (d, *J* = 13.6 Hz, 2H), 7.76 (d, *J* = 8.9 Hz, 2H), 7.64 (t, *J* = 7.7 Hz, 2H), 7.49 (t, *J* = 7.5 Hz, 2H), 6.60 (t, *J* = 12.5 Hz, 2H), 6.49 (d, *J* = 13.7 Hz, 2H), 4.21 (t, *J* = 7.3 Hz, 4H), 2.56 - 2.51 (m, 4H), 1.91 (s, 12H), 1.87 - 1.76 (m, 8H).

HRMS of ICG. HRMS (ESI-TOF) m/z: calcd. for [M + 2H]^+^ C_43_H_49_N_2_O_6_S_2_^+^ = 753.3027; found 753.3033.

### Structural and purity characterization of IR-780

^1^H-NMR spectrum of IR-780 in DMSO. ^1^H NMR (400 MHz, DMSO-*d*_6_) δ 8.28 (d, *J* = 14.1 Hz, 2H), 7.66 (d, *J* = 7.4 Hz, 2H), 7.53 - 7.41 (m, 4H), 7.31 (t, *J* = 7.3 Hz, 2H), 6.37 (d, *J* = 14.2 Hz, 2H), 4.23 (t, *J* = 7.2 Hz, 4H), 2.78 - 2.69 (m, 4H), 1.93 - 1.84 (m, 2H), 1.84 - 1.75 (m, 4H), 1.70 (s, 12H), 0.99 (t, *J* = 7.2 Hz, 6H).

HRMS of IR-780. HRMS (ESI-TOF) m/z: calcd. for [M]^+^ C_36_H_44_ClN_2_^+^ = 539.3188; found 539.3190.

### Structural and purity characterization of IR-780Ac

^1^H-NMR spectrum of IR-780Ac in CDCl_3_. ^1^H NMR (400 MHz, CDCl_3_) δ 8.81 (d, *J* = 13.9 Hz, 2H), 7.32 (d, *J* = 7.4 Hz, 2H), 7.36 - 7.69 (m, 2H), 7.22 - 7.14 (m, 2H), 7.03 (d, *J* = 8.0 Hz, 2H), 6.04 (d, *J* = 14.0 Hz, 2H), 4.44 (s, 1H), 3.98 (t, *J* = 7.5 Hz, 4H), 3.71 - 3.69 (m, 1H), 2.59 - 2.50 (m, 4H), 2.04 - 1.96 (m, 2H), 1.94 (s, 3H), 1.91 - 1.84 (m, 4H), 1.74 (s, 12H), 1.05 (t, *J* = 7.4 Hz, 6H).

HRMS of IR-780Ac. HRMS (ESI-TOF) m/z: calcd. for [M]^+^ C_41_H_52_N_3_O_3_S = 666.3724; found 666.3725.

### ICG-/IR-780-/IR-780Ac-based staining analysis of tissue sections

Specimens embedded in OCT compound were cut into tissue sections (thickness: 15 µm) and attached to glass slides (CITOGLAS). The tissue slides were stored at -20 ^o^C before staining. Subsequently, the tissue slides were stained in a dye solution (50 nM) for 10 min at 37 ^o^C. The tissue slides were washed with dimethyl sulfoxide (DMSO; 3 min) and phosphate-buffered saline-Tween (PBST; 4 min). Finally, the labeled slides were imaged using a scanner (Azure, Sapphire) under the 800 nm channel with certain fluorescence intensity, pixel size 20 µm. ImageJ was used to quantify the signals of images.

### ICG-/IR-780-/IR-780Ac-based histological electrophoresis analysis of tissue sections

Proteins within the frozen sections were labeled and separated using a previously described method. Frozen sections (thickness, 60 μm) were adhered to the array mold (20 × 20, photocrosslinking resin) before lysis and labeling. For simultaneous lysis and labeling, the tissue section adhered to the mold of microwell array was carefully placed to a petri dish (diameter, 5 cm) containing dye solution [5 ml, 1 μM in radio immunoprecipitation assay (RIPA) buffer] and incubated for 10 min at room temperature in the dark. Immediately after lysis and labeling were complete, the mold was carefully placed into uncross-linked stacking gels (with the tissue sections facing upward), which had been poured on top of the cross-linked separating gel in the electrophoresis tank. The stacking gels entered the microwells from the side without tissue sections and cross-linked for 3 min at room temperature to form stacking gel columns. To better separate the proteins of interest in the tissues according to molecular mass, 10% polyacrylamide gel (PAG) was chosen for the 3D histological electrophoresis of tissue sections. Electrophoresis buffer (10 ×, Yeasen, 20319ES76) was carefully poured into the electrophoresis tank until the negative electrode was in complete contact with the liquid surface of electrophoresis buffer to form a current path. After 10 min of running electrophoresis at 30 V, separated proteins in an intact gel could be obtained. Following the 3D histological electrophoresis, the proteins in the tissue were separated with preserving their histological information.

The post-electrophoresis gels containing proteins were fully frozen at -80 °C and then fractionated into layers with a thickness of 600 μm. The fractionated gel layers (fractions 1 to 8) containing the signals of the proteins of interest were then imaged by a NIR scanner (Azure, Sapphire) under the 800 nm channel with a specific fluorescence intensity and pixel size of 100 µm.

### H&E staining analysis

All tissue sections (thickness: 15 µm) were stored at -20 ^o^C until staining. After fixing sections in 4% paraformaldehyde, H&E staining was performed according to the manufacturer's protocol for the H&E kit (Beyotime Institute of Biotechnology, Cat. No. C0105). H&E-stained images of tissues were acquired by using the upright microscope (Nikon eclipse 80i).

### Lysis of tissues

The tissues were cut into sufficiently fine pieces, and an appropriate amount of RIPA buffer was added to an Eppendorf tube containing the tissue pieces. To ensure sufficient lysis, the tube was sonicated for 15 min at 4 ^o^C under a contact-free sonicator with 80% power density. The lysed tissues were then centrifuged for 20 min at 15000 *g* to completely pellet the debris. Finally, the supernatant (lysate) was collected and its concentration was determined by UV-vis.

### ICG, IR-780, and IR-780Ac labeling of protein standards

The labeling of BSA was taken as an example. BSA (100 µM in PBS; Meilunbio, MA0015) was pipetted into an Eppendorf tube, and dye solution (2 mM in DMSO) was added to the Eppendorf tube, ensuring that the molar ratio of dye/BSA was 1:1. Then, the solution was placed in a 60 ^o^C shaker to react for 2 h to obtain the dye-labeled protein samples. β-LG was labeled by ICG, IR-780, and IR-780Ac, respectively following the same protocol.

### Fluorescent probe labeling of tissue lysate

Tissue lysate was diluted to 10 µM with PBS. IR-780 (2 mM in DMSO) was added to the tissue lysate, maintaining a final molar ratio of IR-780/total proteins of 1:1. After reacting at room temperature for 2 h, the proteins in tissue lysate were selectively labeled by IR-780.

### Sodium dodecyl sulfate-polyacrylamide gel electrophoresis (SDS-PAGE) analysis and gel imaging

SDS-PAGE analysis was performed with dedicated SDS-PAGE kits (Yeasen). The dye-labeled tissue lysate was mixed with protein loading buffer (Yeasen, 20315ES05, 5 ×). After sample preparation, electrophoresis buffer was added, and electrophoresis was run at 80 V for the stacking gel and 120 V for the separating gel (American BIO-RAD electrophoresis system). Electrophoresis was terminated when the bromophenol blue was 1 cm from the bottom of the gel. The post-electrophoresis gel membranes were scanned by a scanner (Azure, Sapphire) under the 800 nm channel with a certain fluorescence intensity, and pixel size of 100 µm. ImageJ was used to measure the signals of images.

### Statistical analyses

Results are presented as mean ± standard deviation (SD). Statistical analysis was performed using PRISM 8 graphing software (GraphPad) and Origin 2024b. For all statistical tests, *P* values < 0.05 were considered significant. Quantified similarity was calculated using MATLAB R2021a.

## Supplementary Material

Supplementary figures and table: Collection of clinical specimens and information; experimental protocol of the ICG-/IR-780-/IR-780Ac-based staining analysis of tissue sections; experimental protocol of the ICG-/IR-780-/IR-780Ac-based histological electrophoresis analysis of tissue sections; experimental protocol of H&E staining; experimental protocol of tissues lysis; experimental protocol of the ICG, IR-780, and IR-780Ac labeling of protein standards; experimental protocol of tissue lysate labeling; the complete process of SDS-PAGE analysis and gel imaging.

## Abbreviations

3D: three-dimensional
FDA: the US Food and Drug Administration
NIR: near infrared
ICG: indocyanine green
FGS: fluorescence-guided surgery
FLT: fluorescence lifetime
TME: tumor microenvironment
TSD-HE: tumor-selective dye-based histological electrophoresis
BSA: bovine serum albumin
β-LG: β-lactoglobulin
H&E: hematoxylin & eosin
ROC: receiver operating characteristic
AUC: area under the curve
AI: artificial intelligence
OCT: optimal cutting temperature
DMSO: dimethyl sulfoxide
PBST: phosphate-buffered saline-Tween
RIPA: radio immunoprecipitation assay
PAG: polyacrylamide gel
SDS-PAGE: sodium dodecyl sulfate-polyacrylamide gel electrophoresis
SD: standard deviation

## References

[1] Yu K, Hu V, Wang F, Matulonis UA, Mutter GL, Golden JA (2020). Deciphering serous ovarian carcinoma histopathology andplatinum response by convolutional neural networks. BMC Med.

[2] Marostica E, Barber R, Denize T, Kohane IS, Signoretti S, Golden JA (2021). Development of a histopathology informatics pipeline for classification and prediction of clinical outcomes in subtypes of renal cell carcinoma. Clin Cancer Res.

[3] Wulczyn E, Steiner DF, Moran M, Plass M, Reihs R, Tan F (2021). Interpretable survival prediction for colorectal cancer using deep learning. NPJ Digit Med.

[4] Chuang W, Chen C, Yu W, Yeh C, Chang S, Ueng S (2021). Identification of nodal micrometastasis in colorectal cancer using deep learning on annotation-free whole-slide images. Mod Pathol.

[5] Kleppe A, Skrede O, Raedt S, Liestøl K, Kerr DJ, Danielsen HE (2021). Designing deep learning studies in cancer diagnostics. Nat Rev Cancer.

[6] Zech JR, Badgeley MA, Liu M, Costa AB, Titano JJ, Oermann EK (2018). Variable generalization performance of a deep learning model to detect pneumonia in chest radiographs: a cross-sectional study. PLoS Med.

[7] Narla A, Kuprel B, Sarin K, Novoa R, Ko J (2018). Automated classification of skin lesions: from pixels to practice. J Invest Dermatol.

[8] Winkler JK, Fink C, Toberer F, Enk A, Deinlein T, Hofmann-Wellenhof R (2019). Association between surgical skin markings in dermoscopic images and diagnostic performance of a deep learning convolutional neural network for melanoma recognition. JAMA Dermatol.

[9] Mieog JSD, Achterberg FB, Zlitni A, Hutteman M, Burggraaf J, Swijnenburg RJ (2022). Fundamentals and developments in fluorescence-guided cancer surgery. Nat Rev Clin Oncol.

[10] Lee JYK, Cho SS, Stummer W, Tanyi JL, Vahrmeijer AL, Rosenthal E (2019). Review of clinical trials in intraoperative molecular imaging during cancer surgery. J Biomed Opt.

[11] Lauwerends LJ, Driel P, Baatenburg de Jong RJ, Hardillo JAU, Koljenovic S, Puppels G (2021). Real-time fluorescence imaging in intraoperative decision making for cancer surgery. Lancet Oncol.

[12] Schaafsma BE, Mieog JS, Hutteman M, van der Vorst JR, Kuppen PJ, Löwik CW (2011). The clinical use of indocyanine green as a near-infrared fluorescent contrast agent for image-guided oncologic surgery. J Surg Oncol.

[13] Barabino G, Klein JP, Porcheron J, Grichine A, Coll JL, Cottier M (2016). Intraoperative near-infrared fluorescence imaging using indocyanine green in colorectal carcinomatosis surgery: proof of concept. Eur J Surg Oncol.

[14] Liberale G, Bourgeois P, Larsimont D, Moreau M, Donckier V, Ishizawa T (2017). Indocyanine green fluorescence-guided surgery after IV injection in metastatic colorectal cancer: a systematic review. Eur J Surg Oncol.

[15] Zeh R, Sheikh S, Xia L, Pierce J, Newton A, Predina J (2017). The second window ICG technique demonstrates a broad plateau period for near infrared fluorescence tumor contrast in glioblastoma. PLoS One.

[16] Lee JY, Thawani JP, Pierce J, Zeh R, Martinez-Lage M, Chanin M (2016). Intraoperative near-infrared optical imaging can localize gadolinium-enhancing gliomas during surgery. Neurosurgery.

[17] Yokoyama J, Fujimaki M, Ohba S, Anzai T, Yoshii R, Ito S (2013). A feasibility study of NIR fluorescent image-guided surgery in head and neck cancer based on the assessment of optimum surgical time as revealed through dynamic imaging. Onco Targets Ther.

[18] Veys I, Pop CF, Barbieux R, Moreau M, Noterman D, de Neubourg F (2018). ICG fluorescence imaging as a new tool for optimization of pathological evaluation in breast cancer tumors after neoadjuvant chemotherapy. PLoS One.

[19] Nicoli F, Saleh DB, Baljer B, Chan CD, Beckingsale T, Ghosh KM (2021). Intraoperative near-infrared fluorescence (NIR) imaging with indocyanine green (ICG) can identify bone and soft tissue sarcomas which may provide guidance for oncological resection. Ann Surg.

[20] Brookes MJ, Chan CD, Nicoli F, Crowley TP, Ghosh KM, Beckingsale T (2021). Intraoperative near-infrared fluorescence guided surgery using indocyanine green (ICG) for the resection of sarcomas may reduce the positive margin rate: an extended case series. Cancers.

[21] Kedrzycki MS, Leiloglou M, Chalau V, Chiarini N, Thiruchelvam PTR, Hadjiminas DJ (2021). The impact of temporal variation in indocyanine green administration on tumor identification during fluorescence guided breast surgery. Ann Surg Oncol.

[22] Onda N, Kimura M, Yoshida T, Shibutani M (2016). Preferential tumor cellular uptake and retention of indocyanine green for in vivo tumor imaging. Int J Cancer.

[23] Mc Larney BE, Sonay AY, Apfelbaum E, Mostafa N, Monette S, Goerzen D (2024). A pan-cancer dye for solid-tumour screening, resection and wound monitoring via short-wave and near-infrared fluorescence imaging. Nat Biomed Eng.

[24] Gao RW, Teraphongphom N, de Boer E, van den Berg NS, Divi V, Kaplan MJ (2018). Safety of panitumumab-IRDye800CW and cetuximab-IRDye800CW for fluorescence-guided surgical navigation in head and neck cancers. Theranostics.

[25] Boogerd LSF, Hoogstins CES, Schaap DP, Kusters M, Handgraaf HJM, van der Valk MJM (2018). Safety and effectiveness of SGM-101, a fluorescent antibody targeting carcinoembryonic antigen, for intraoperative detection of colorectal cancer: a dose-escalation pilot study. Lancet Gastroenterol Hepatol.

[26] Samkoe KS, Sardar HS, Bates BD, Tselepidakis NN, Gunn JR, Hoffer-Hawlik KA (2019). Preclinical imaging of epidermal growth factor receptor with ABY-029 in soft-tissue sarcoma for fluorescence-guided surgery and tumor detection. J Surg Oncol.

[27] Hoogstins CE, Tummers QR, Gaarenstroom KN, de Kroon CD, Trimbos JB, Bosse T (2016). A novel tumor-specific agent for intraoperative near-infrared fluorescence imaging: a translational study in healthy volunteers and patients with ovarian cancer. Clin Cancer Res.

[28] Shen D, Xu B, Liang K, Tang R, Sudlow GP, Egbulefu C (2020). Selective imaging of solid tumours via the calcium-dependent high-affinity binding of a cyclic octapeptide to phosphorylated Annexin A2. Nat Biomed Eng.

[29] Choi HS, Gibbs SL, Lee JH, Kim SH, Ashitate Y, Liu F (2013). Targeted zwitterionic near-infrared fluorophores for improved optical imaging. Nat Biotechnol.

[30] Schupper AJ, Baron RB, Cheung W, Rodriguez J, Kalkanis SN, Chohan MO (2022). 5-Aminolevulinic acid for enhanced surgical visualization of high-grade gliomas: a prospective, multicenter study. J Neurosurg.

[31] Veiseh M, Gabikian P, Bahrami SB, Veiseh O, Zhang M, Hackman RC (2007). Tumor paint: a chlorotoxin:Cy5.5 bioconjugate for intraoperative visualization of cancer foci. Cancer Res.

[32] Smith BL, Gadd MA, Lanahan CR, Rai U, Tang R, Rice-Stitt T (2018). Real-time, intraoperative detection of residual breast cancer in lumpectomy cavity walls using a novel cathepsin-activated fluorescent imaging system. Breast Cancer Res Treat.

[33] Weissleder R, Tung CH, Mahmood U, Bogdanov A (1999). In vivo imaging of tumors with protease-activated near-infrared fluorescent probes. Nat Biotechnol.

[34] Pringle TA, Chan CD, Luli S, Blair HJ, Rankin KS, Knight JC (2022). Synthesis and in vivo evaluation of a site-specifically labeled radioimmunoconjugate for dual-modal (PET/NIRF) imaging of MT1-MMP in sarcomas. Bioconjug Chem.

[35] Pétusseau A, Bruza P, Pogue B (2022). Protoporphyrin IX delayed fluorescence imaging: a modality for hypoxia-based surgical guidance. J Biomed Opt.

[36] Chen T, Chen Z, Liu R, Zheng S (2019). A NIR fluorescent probe for detection of viscosity and lysosome imaging in live cells. Org Biomol Chem.

[37] Song Y, Zhang H, Wang X, Geng X, Sun Y, Liu J (2021). One stone, three birds: pH triggered transformation of aminopyronine and iminopyronine based lysosome targeting viscosity probe for cancer visualization. Anal Chem.

[38] Zhang J, Rakhimbekova A, Duan X, Yin Q, Foss CA, Fan Y (2021). A prostate-specific membrane antigen activated molecular rotor for real-time fluorescence imaging. Nat Commun.

[39] Zhao T, Huang G, Li Y, Yang S, Ramezani S, Lin Z (2016). A transistor-like pH nanoprobe for tumour detection and image-guided surgery. Nat Biomed Eng.

[40] Voskuil FJ, Steinkamp PJ, Zhao T, van der Vegt B, Koller M, Doff JJ (2020). Exploiting metabolic acidosis in solid cancers using a tumor-agnostic pH-activatable nanoprobe for fluorescence-guided surgery. Nat Commun.

[41] Kokudo N, Ishizawa T (2012). Clinical application of fluorescence imaging of liver cancer using indocyanine green. Liver Cancer.

[42] Pogue BW, Rosenthal EL, Achilefu S, van Dam GM (2018). Perspective review of what is needed for molecular-specific fluorescence-guided surgery. J Biomed Opt.

[43] Pal R, Lwin TM, Krishnamoorthy M, Collins HR, Chan CD, Prilutskiy A (2023). Fluorescence lifetime of injected indocyanine green as a universal marker of solid tumours in patients. Nat Biomed Eng.

[44] Chen F, Chandrashekar DS, Varambally S, Creighton CJ (2019). Pan-cancer molecular subtypes revealed by mass-spectrometry-based proteomic characterization of more than 500 human cancers. Nat Commun.

[45] Akbani R, Ng PK, Werner HM, Shahmoradgoli M, Zhang F, Ju Z (2014). A pan-cancer proteomic perspective on The Cancer Genome Atlas. Nat Commun.

[46] Wang D, Eraslan B, Wieland T, Hallström B, Hopf T, Zolg DP (2019). A deep proteome and transcriptome abundance atlas of 29 healthy human tissues. Mol Syst Biol.

[47] Lawrence RT, Villén J (2014). Drafts of the human proteome. Nat Biotechnol.

[48] Wilhelm M, Schlegl J, Hahne H, Gholami AM, Lieberenz M, Savitski MM (2014). Mass-spectrometry-based draft of the human proteome. Nature.

[49] Nusinow DP, Szpyt J, Ghandi M, Rose CM, McDonald ER, Kalocsay M (2020). Quantitative proteomics of the cancer cell line encyclopedia. Cell.

[50] Wang Y, Zhang F, Jia Y, Han T, Zhang C, Qu L (2024). Tumor receptor-seeking dyes for rapid intraoperative definition of tumor margin and histopathological morphology. CCS Chem.

[51] Zhang F, Xu J, Yue Y, Wang Y, Sun J, Song D (2023). Three-dimensional histological electrophoresis enables fast automatic distinguishment of cancer margins and lymph node metastases. Sci Adv.

[52] Zhang F, Xu J, Zhang C, Li Y, Gao J, Qu L (2023). Three-dimensional histological electrophoresis for high-throughput cancer margin detection in multiple types of tumor specimens. Nano Lett.

[53] Tian R, Feng X, Wei L, Dai D, Ma Y, Pan H (2022). A genetic engineering strategy for editing near-infrared-II fluorophores. Nat Commun.

[54] Canovas C, Bellaye PS, Moreau M, Romieu A, Denat F, Goncalves V (2018). Site-specific near-infrared fluorescent labelling of proteins on cysteine residues with meso-chloro-substituted heptamethine cyanine dyes. Org Biomol Chem.

[55] Usama SM, Burgess K (2021). Hows and whys of tumor-seeking dyes. Acc Chem Res.

[56] Bai L, Hu Z, Han T, Wang Y, Xu J, Jiang G, Feng X (2022). Super-stable cyanine@albumin fluorophore for enhanced NIR-II bioimaging. Theranostics.

[57] Usama SM, Park GK, Nomura S, Baek Y, Choi HS, Burgess K (2020). Role of albumin in accumulation and persistence of tumor-seeking cyanine dyes. Bioconjug Chem.

[58] Thavornpradit S, Usama SM, Park GK, Shrestha JP, Nomura S, Baek Y (2019). QuatCy: a heptamethine cyanine modification with improved characteristics. Theranostics.

[59] Du Y, Xu J, Han T, Jiang Z, Zhang Y, Li J (2024). Albumin-seeking dyes with adjustable assemblies in situ enable programmable imaging windows and targeting tumor imaging. Theranostics.

[60] Karsch-Bluman A, Feiglin A, Arbib E, Stern T, Shoval H, Schwob O (2019). Tissue necrosis and its role in cancer progression. Oncogene.

[61] Yamamoto A, Huang Y, Krajina BA, McBirney M, Doak AE, Qu S (2023). Metastasis from the tumor interior and necrotic core formation are regulated by breast can-cer-derived angiopoietin-like 7. Proc Natl Acad Sci U S A.

[62] Lemos MB, Okoye E (2019). Atlas of surgical pathology grossing. Springer International Publishing.

