# Supplementary material for: Tumor-selective dye-based histological electrophoresis enables intraoperative tumor diagnosis via tumor-specific enhancement
Source: Theranostics. 2025 Jan 6;15(5):2052–68. doi: 10.7150/thno.105500 (PMC11780519; doi:10.7150/thno.105500)
Supplement: Supplementary file 1 — Supplementary figures and table: Collection of clinical specimens and information; experimental protocol of the ICG-/IR-780-/IR-780Ac-based staining analysis of tissue sections; experimental protocol of the ICG-/IR-780-/IR-780Ac-based histological electrophoresis analysis of tissue sections; experimental protocol of H&E staining; experimental protocol of tissues lysis; experimental protocol of the ICG, IR-780, and IR-780Ac labeling of protein standards; experimental protocol of tissue lysate labeling; the complete process of SDS-PAGE analysis and gel imaging. [file thnov15p2052s1.pdf]

Supplementary material

Tumor-selective dye-based histological electrophoresis enables intraoperative tumor diagnosis via tumor-specific enhancement

Feiran Zhang,[1,2] Jianing Cheng,[1,2] Xu Peng,[1,3] Chengbin Zhang,[4] Limei Qu,[4]* Songling Zhang,[5]* Junhu Zhang,[1,2]* Shoujun Zhu[1,2]*

Supplementary Figures and Tables

Table S1. Clinical information of the tumors in our patient cohort.

Patient ID	Gender	Age, years	T stage	N stage
**Liver cancer**				
G0375	Male	58	T2	NX
S0002	Female	53	T1	
G0211	Female	44	T3	
G1952	Male	43	T3	
G1753	Male	55	T2	NX
G0268	Male	38	T2	NX
G0238	Male	54	T1	
G0163	Male	65	T3	
G1630	Male	48	T1	NX
**Cholangiocarcinoma**				
G0193	Male	48	T2	
G0329	Female	65	Tis	N0
G0235	Female	47	T3	N0
G0065	Male	57	T2	N0
G0006	Male	73	T2	NX
S0003	Female	73	T2	N0
**Pancreatic neuroendocrine tumor**				
G0534	Female	47		
**Pancreatic cancer**				
G1631	Male	66	T2	N0
G1533	Male	74	T2	N0
G1172	Male	79	T3	N0
G0241	Male	44	T3	N1
G0223	Male	64	T2	N0
G2059	Female	65	T2	
S0006	Male	66	T2	N0
**Cervical cancer**				
F0076	Female	40	T1	
F0001	Female	57	T1	N0
F0012	Female	39	T1	N0
F0112	Female	57	T1	N1
**Breast cancer**				
R0010	Female	67	T1	N2
R0011	Female	52	T2	N1
R0012	Female	45	T1	N0
R0013	Female	38	T1	N1
**Esophageal carcinoma**				
S0007	Male	46	T1	N0
S0008	Male	52	T2	N0
S0009	Female	63	T1	N1

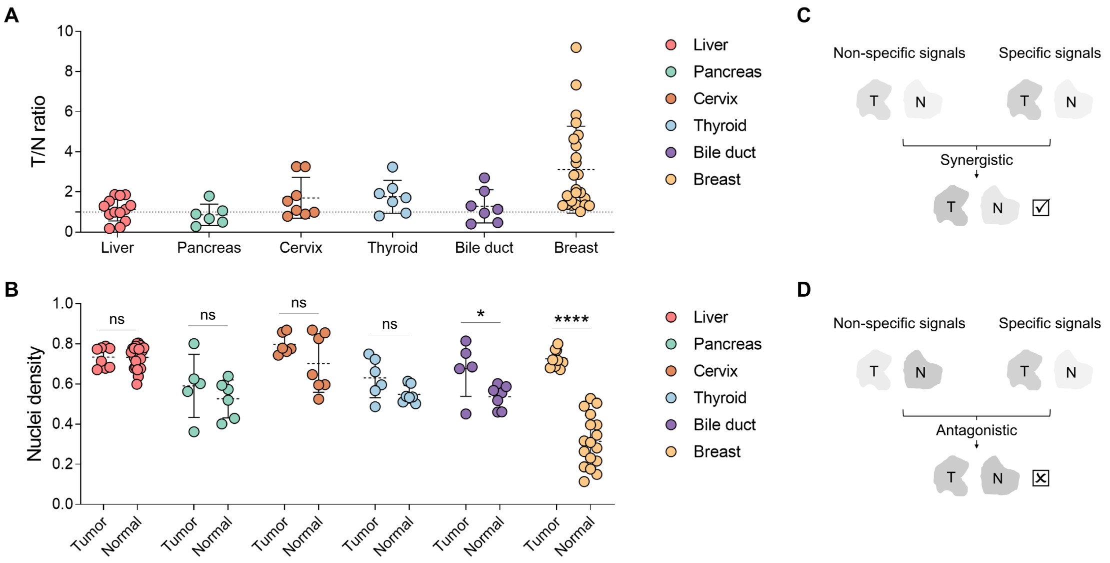

Figure S1. Comparison of tumor-selective dye-based direct staining approach for the diagnosis of malignant regions in the tissue sections of different cancer specimens. (A) Tumor-to-normal tissue ratios in six cancer specimens via direct staining approach. (B) Comparison of nuclei density between tumor and normal regions six cancer specimens evaluated by H&E images. Statistical significance is calculated using a *t*-test: **P* = 0.0348 and *****P* < 0.0001. (C and D) Schematic representation illustrating that the synergistic (C) and antagonistic (D) relationship between specific and non-specific signals can influence the diagnostic results of the direct staining approach.

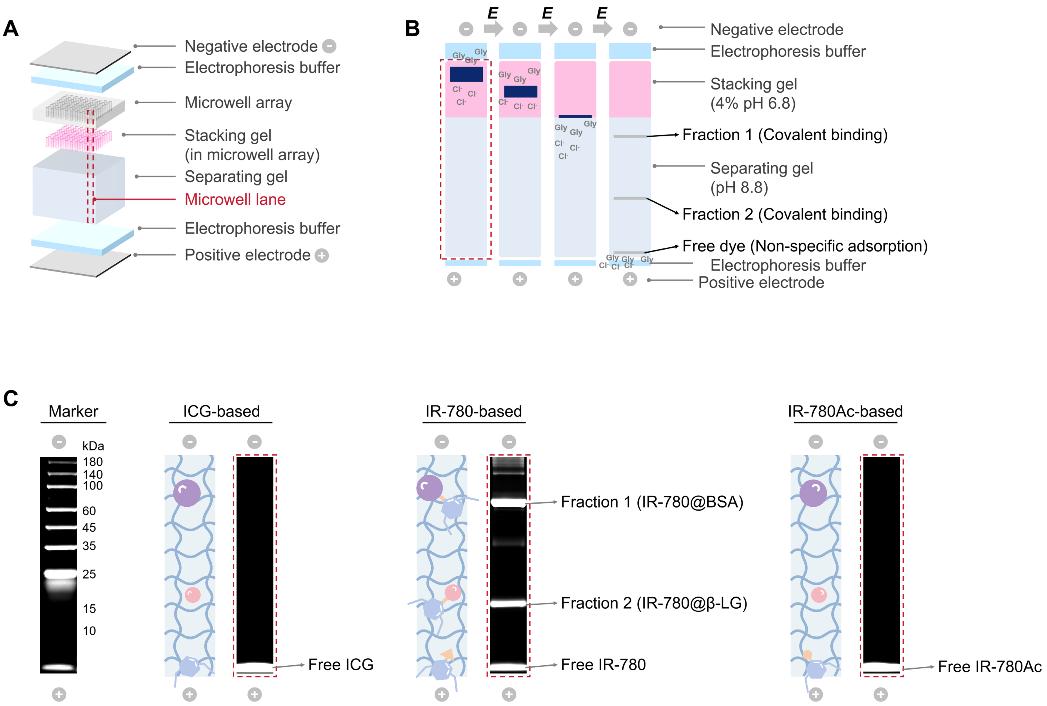

Figure S2. Mechanism of 3D histological electrophoresis for recognizing covalent signals while preserving histological information. (A) Structure of the 3D histological electrophoresis device equipped with a negative electrode, a stacking gel with a microwell array, a separating gel, and a positive electrode. The microwell array is designed to preserve the histological information of post-electrophoresis signals. Each microwell represented one electrophoresis lane. (B) Mechanism of 3D histological electrophoresis for protein separation in each lane. (C) Comparison of ICG-/IR-780-/IR-780Ac-based electrophoresis analysis for protein standards.

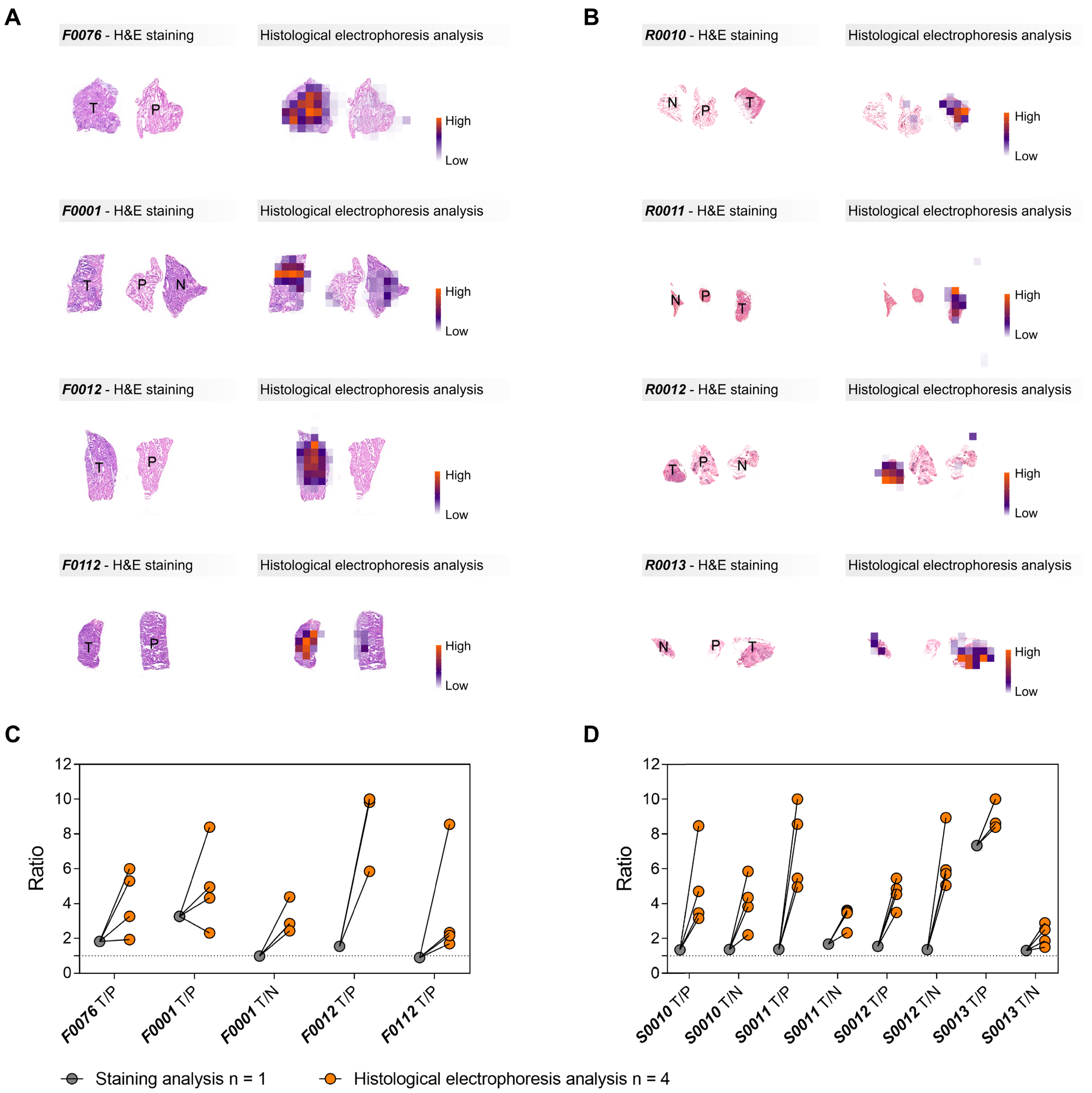

Figure S3. IR-780-based histological analysis in cervical and breast cancer specimens. (A and B) IR-780-based histological electrophoresis analysis in cervical cancer specimens (from four patients F0076, F0001, F0012, and F0112, **Table S1**; A) and breast cancer specimens (from four patients R0010, R0011, R0012, and R0013, **Table S1**; B). Heat map describes histological electrophoresis analysis results. The co-localization of H&E staining results with histological electrophoresis analysis results in tissue sections describes the spatial distribution and abundance of IR-780 covalently bound proteins in tissue sections. The heat map represents the total signal of protein fractions after histological electrophoresis separation (fraction 1 to 8). (C and D) Enhancement of tumor-to-paracancerous (or normal) tissue ratios in cervical (C) and breast (D) cancer via histological electrophoresis analysis.

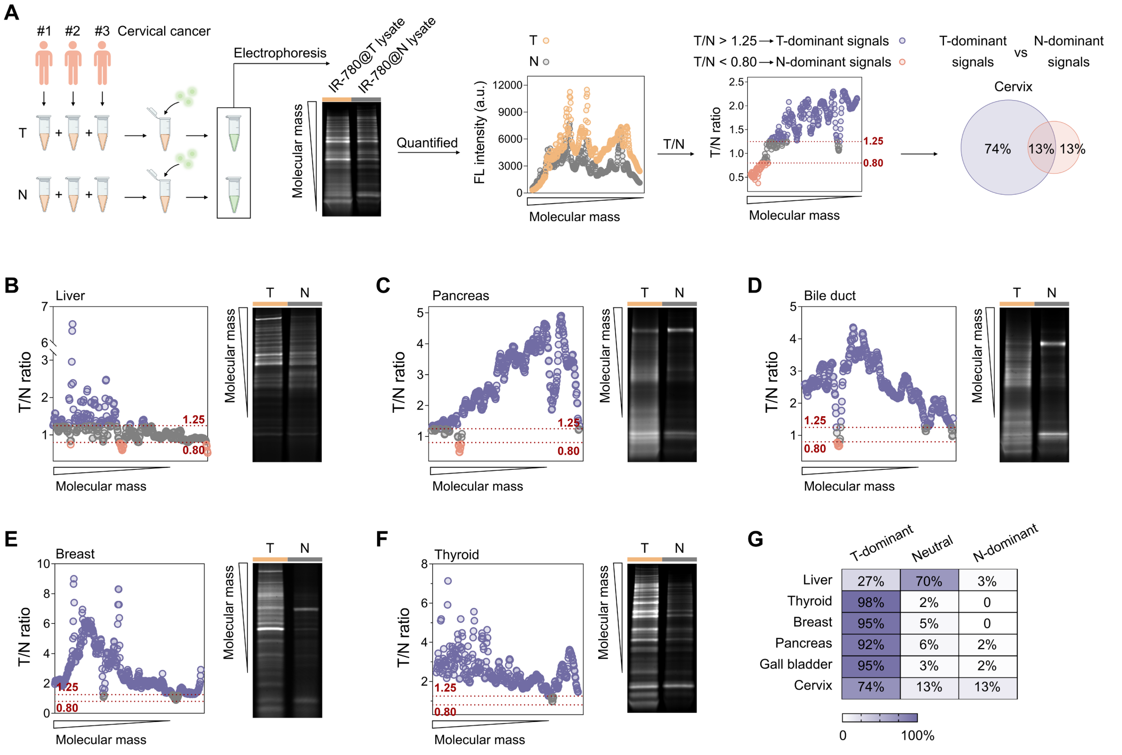

Figure S4. Quantification of the tumor dominant signals after IR-780 labeling for tumor identification. (A) Schematic of the workflow, including tissue lysing and labeling, electrophoresis analysis, and the quantification of tumor tissue dominant and normal tissue dominant signals. Cervical tumors are taken as an example. We define a signal with a tumor-to-normal ratio greater than 1.25 as the tumor tissue dominant signal, while a ratio less than 0.80 is defined as the normal tissue dominant signal. This quantification helps determine the proportion of the signal available for tumor identification (tumor tissue dominant signal) within the overall molecular mass distribution. (B-F) Quantified IR-780-labeled protein signals of tumor and normal tissues in liver (B), pancreas (C), bile duct (D), breast (E), and thyroid (F). (G) Comparison of tumor tissue dominant, neutral, and normal tissue-dominant signals from six types of tumor. In the overall molecular mass distribution, the tumor dominant signal distribution is more extensive than that of normal tissue.

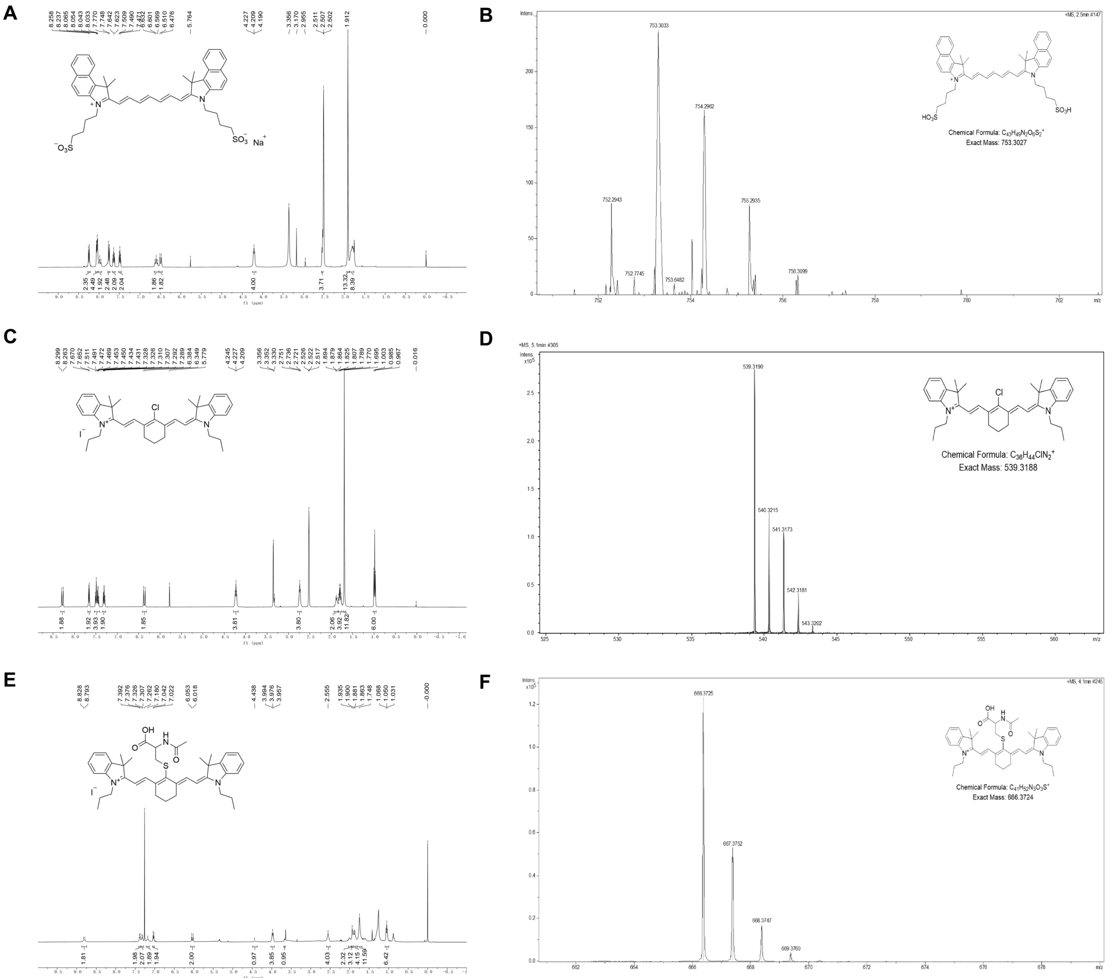

Figure S5. Structural and purity characterization of ICG, IR-780, and IR-780Ac. (A) ^{1}H-NMR spectrum of ICG in DMSO. (B) HRMS of ICG. (C) ^{1}H-NMR spectrum of IR-780 in DMSO. (D) HRMS of IR-780. (E) ^{1}H-NMR spectrum of IR-780Ac in $CDCl_3$. (F) HRMS of IR-780Ac.

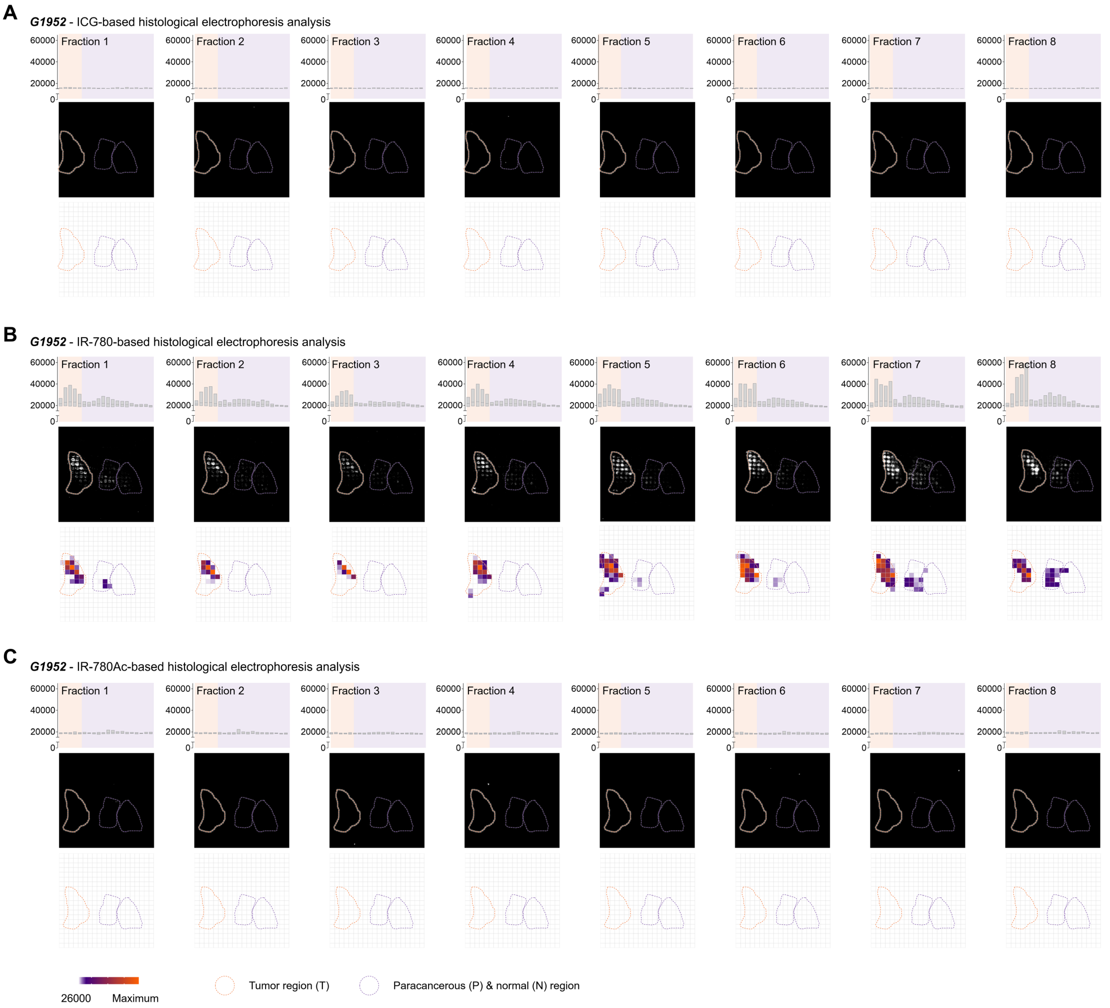

Figure S6. The quantified signal intensity of fractions 1 to 8 after the ICG-/IR-780-/IR-780Ac-based histological electrophoresis analysis for G1952. Comparison of ICG-based (A), IR-780-based (B), and IR-780Ac-based (C) histological electrophoresis analysis of liver cancer specimens (G1952). **Data Note:** The total heat maps of the post-electrophoresis protein signals of fractions 1 to 8 are mentioned in **Figure 2E** (ICG-/IR-780-/IR-780Ac-based histological electrophoresis analysis for G1952).

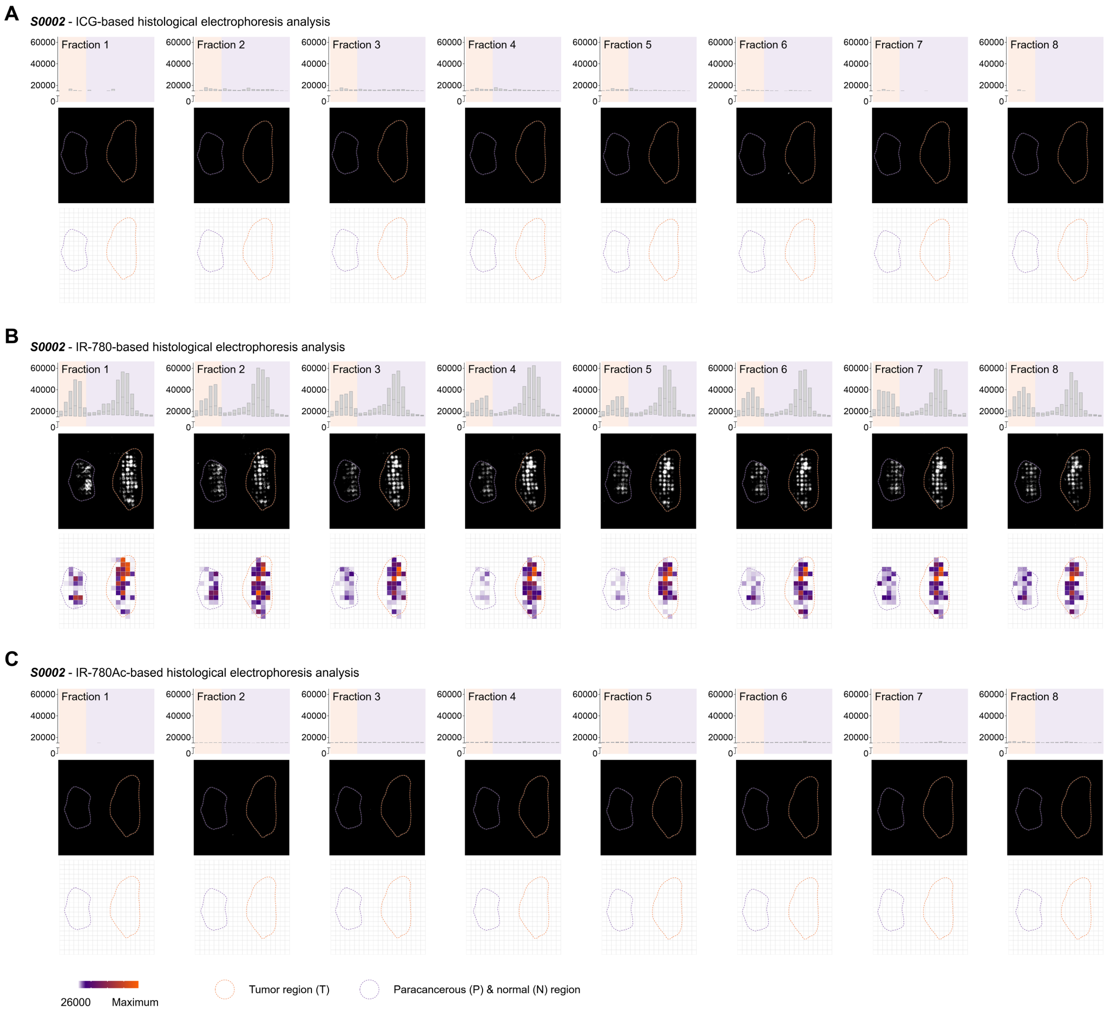

Figure S7. The quantified signal intensity of fractions 1 to 8 after the ICG-/IR-780-/IR-780Ac-based histological electrophoresis analysis for S0002. Comparison of ICG-based (A), IR-780-based (B), and IR-780Ac-based (C) histological electrophoresis analysis of liver cancer specimens (S0002). **Data Note:** The total heat maps of the post-electrophoresis protein signals of fractions 1 to 8 are mentioned in **Figure 4A** (IR-780-based histological electrophoresis analysis for S0002).

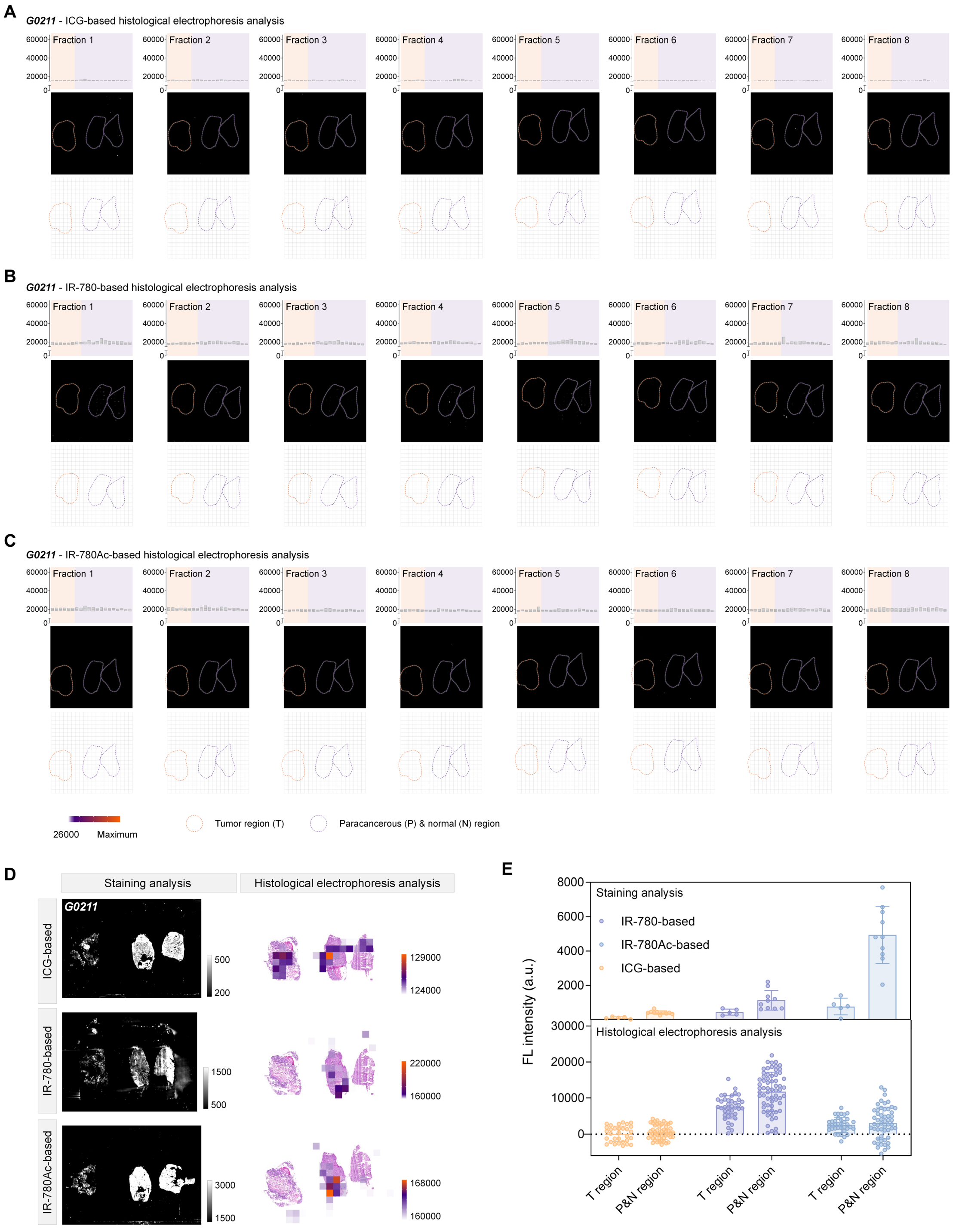

Figure S8. Necrotic tumor tissue lacks the characteristic proteins that can be selectively covalent bound by IR-780. (A-C) Comparison of ICG-based (A), IR-780-based (B), and IR-780Ac-based (C) histological electrophoresis analysis of liver cancer specimens (G0211). (D) Comparison of ICG-/IR-780-/IR-780Ac-based staining analysis and ICG-/IR-780-/IR-780Ac-based histological electrophoresis analysis in the liver cancer specimens (G0211). The tumor regions within the tissue sections of G0211 are histologically confirmed as necrotic tumor tissue. (E) Plots of the signal intensity of multiple ROIs in tumor region and paracancerous and normal

region after ICG-/IR-780-/IR-780Ac-based staining analysis (The number of ROIs is five, and the size of ROIs is 1 mm × 1 mm) and ICG-/IR-780-/IR-780Ac-based histological electrophoresis analysis (ROIs: n > 10).

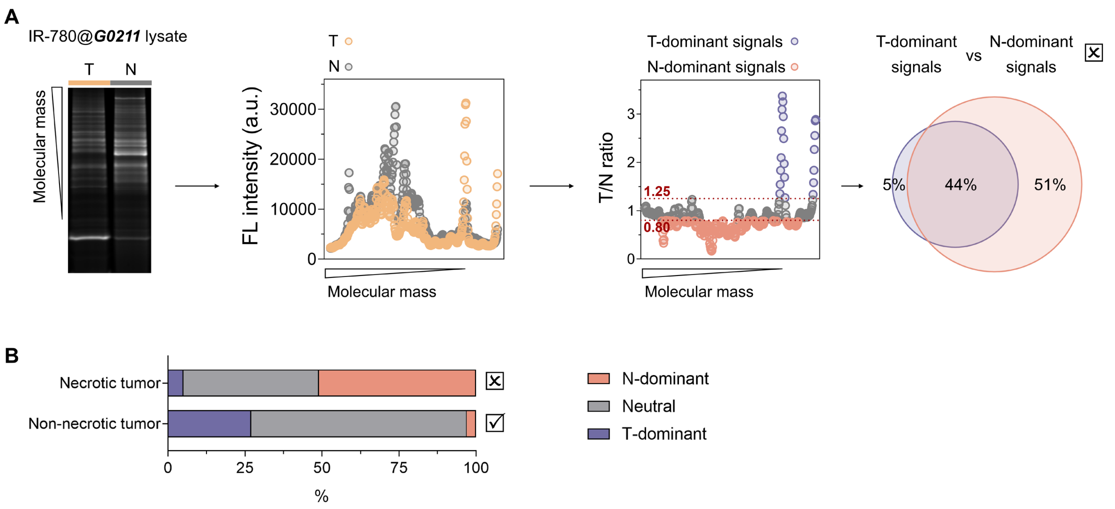

Figure S9. Quantification of the tumor-dominant signals of necrotic tumor after IR-780 labeling. (A) Schematic of the workflow for analyzing tumor dominant signals (5%) and normal tissue dominant signals (51%) in a necrotic tumor (G0211). (B) Comparison of quantified signals between necrotic and non-necrotic tumors, demonstrating a decrease in the protein content inside the necrotic tumors.

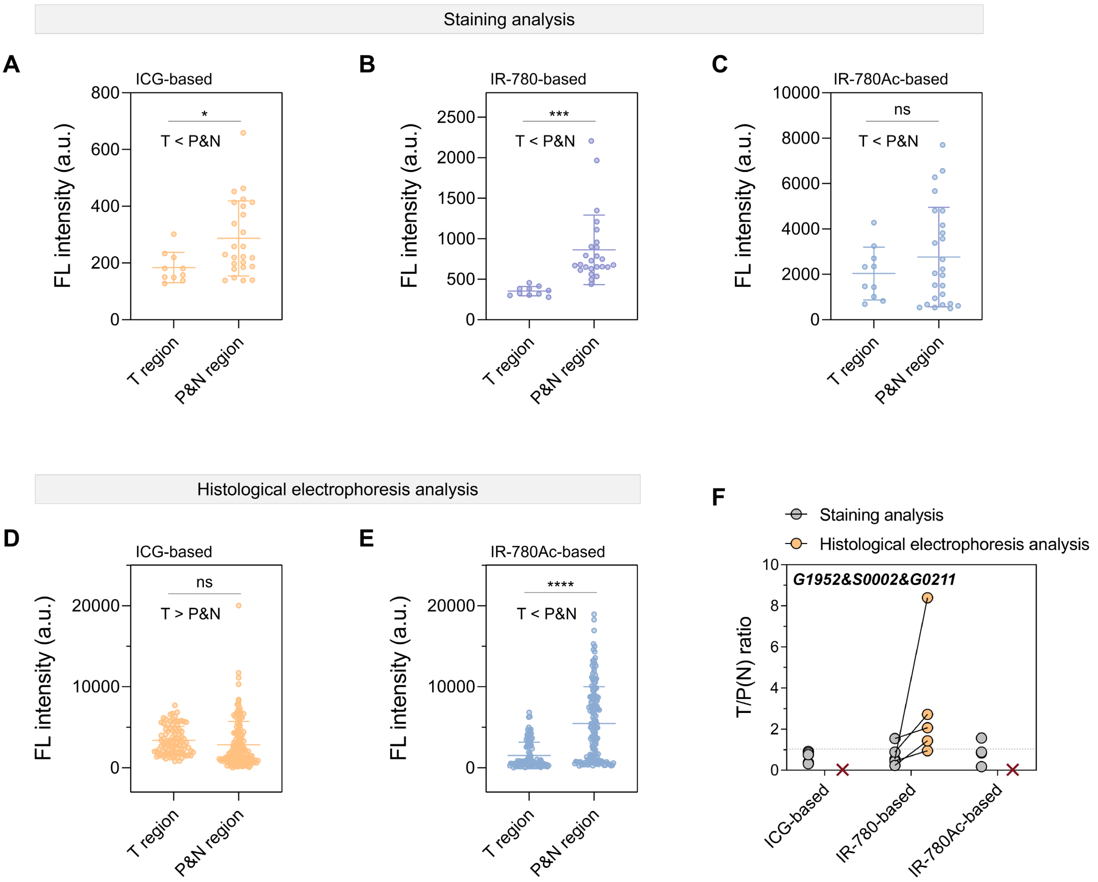

Figure S10. Comparison of the effects of tumor necrosis on staining analysis and histological electrophoresis analysis (A-C) Plot of the signal intensity of ROIs in tumor region and paracancerous and normal region after ICG-based (A), IR-780-based (B) and IR-780Ac-based (C) staining analysis after excluding signals of the necrotic region from G0211. Statistical significance is calculated using a *t*-test: **P* = 0.0244, ****P* = 0.0008, and ns *P* > 0.05. (D and E) Plot of the signal intensity of ROIs in tumor region and paracancerous and normal region after ICG-based (D) and IR-780Ac-based (E) histological electrophoresis analysis after excluding signals of the necrotic region from G0211. Statistical significance is calculated using a *t*-test: ns *P* > 0.05, *****P* < 0.0001. (F) Ratios of the fluorescence intensity collected from the tumor regions to that collected from paracancerous (or normal) tissue regions in one tissue section. For staining analysis, ratios are quantified from fluorescence intensity images. For histological electrophoresis analysis, ratios are quantified from heat maps.

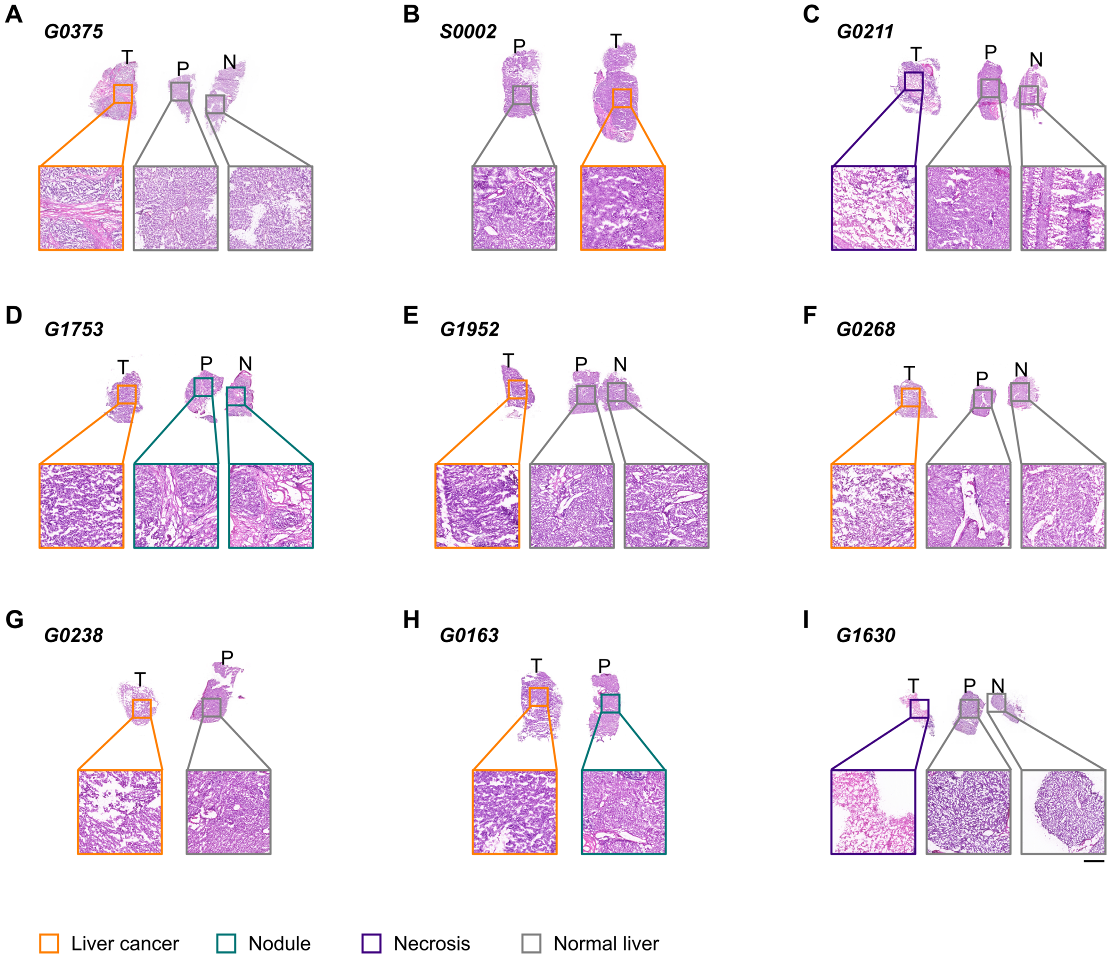

Figure S11. H&E staining results of the liver cancer tumor and the corresponding paracancerous and normal tissues. (A-I) The H&E staining results of G0375 (A), S0002 (B), G0211 (C), G1753 (D), G1952 (E), G0268 (F), G0238 (G), G0163 (H), and G1630 (I) are used to confirm the histological type of the tumor, paracancerous, and normal tissue regions within tissue sections. Scale bar: 500 µm.

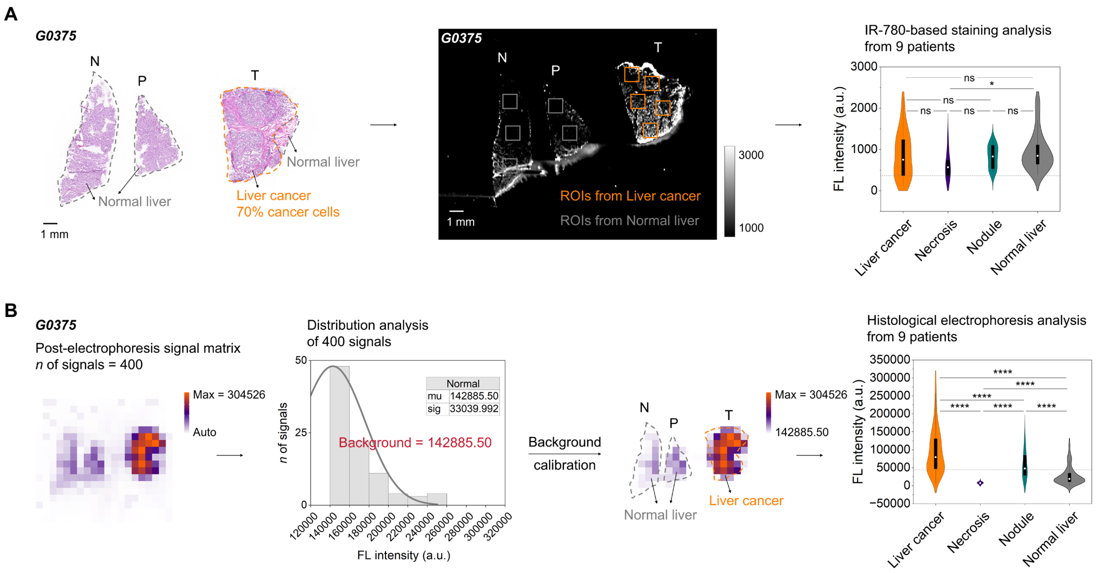

Figure S12. Statistical workflow of staining analysis results and histological electrophoresis analysis results. G0375 is taken as an example. **Data Note:** Here we focus on introducing the methods of analyzing data. All diagnostic reported results for the two strategies have been mentioned above.

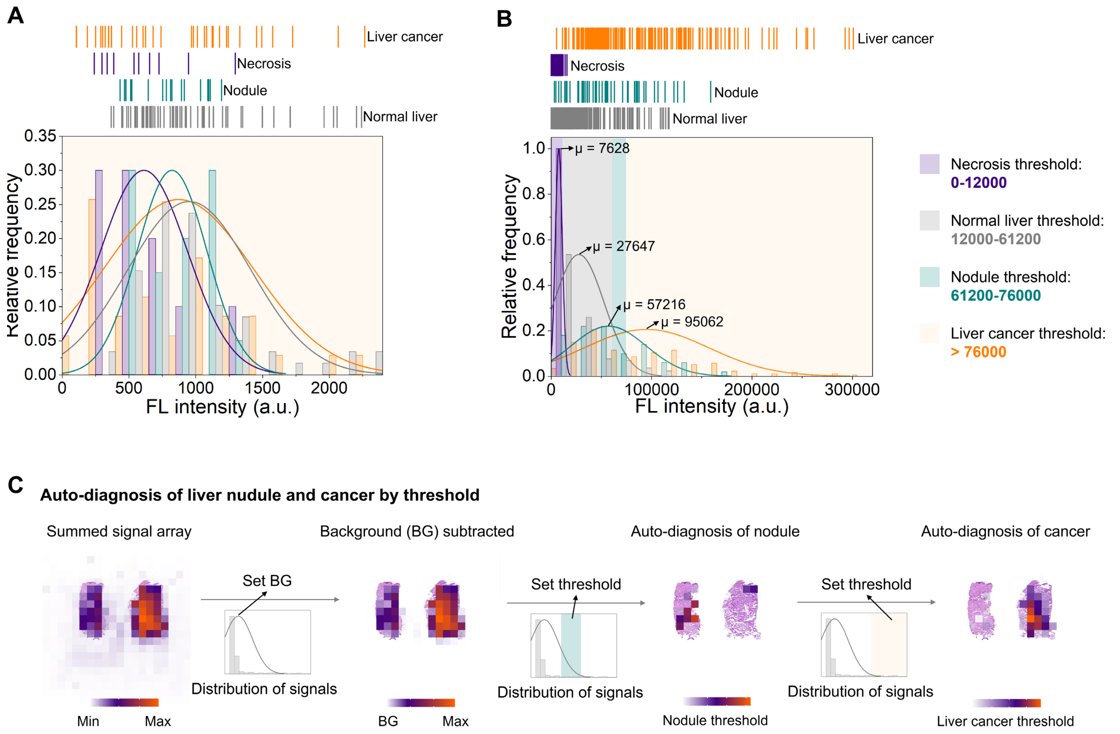

Figure S13. Covalent signal threshold for diagnosing different histological types within liver cancer tissue sections. Histological types within the tissue sections from liver cancer patients include: liver cancer, necrosis, nodule, and normal liver. (A) Histograms of signals after IR-780-based staining analysis. (B) Histograms of signals after IR-780-based histological electrophoresis analysis. (C) Automated diagnosis of the nodule region and cancer region in a liver cancer tissue section (from G0163) using a signal threshold.

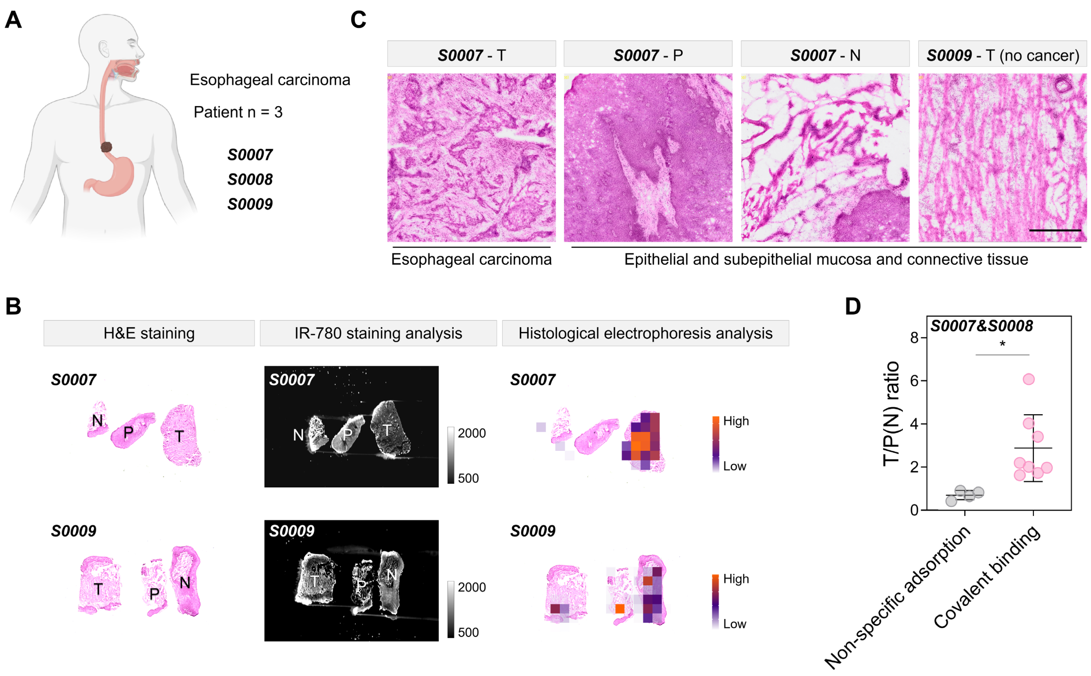

Figure S14. Distinguishing tumors from normal tissues in esophageal carcinoma surgical specimens using histological electrophoresis analysis. (A) Schematic representation of the esophageal carcinoma specimens from three patients (S0007, S0008, and S0009, **Table S1**) to be analyzed. (B) Comparison of IR-780-based staining analysis and IR-780-based histological analysis in the esophageal carcinoma specimens from S0007 and S0009. (C) H&E staining results reflect representative histological types (esophageal carcinoma and epithelial and subepithelial mucosa and connective tissue) in the surgically resected tissue of esophageal carcinoma. Histologically confirmed absence of esophageal carcinoma in the tumor, paracancerous, and normal tissues obtained from S0009. Scale bar: 500 μm. (D) Comparison of the ratios calculated from IR-780-staining strategy and IR-780-based electrophoresis separating strategy. Significant differences are observed between the covalent binding group and the non-specific adsorption group (*$P = 0.0209$).

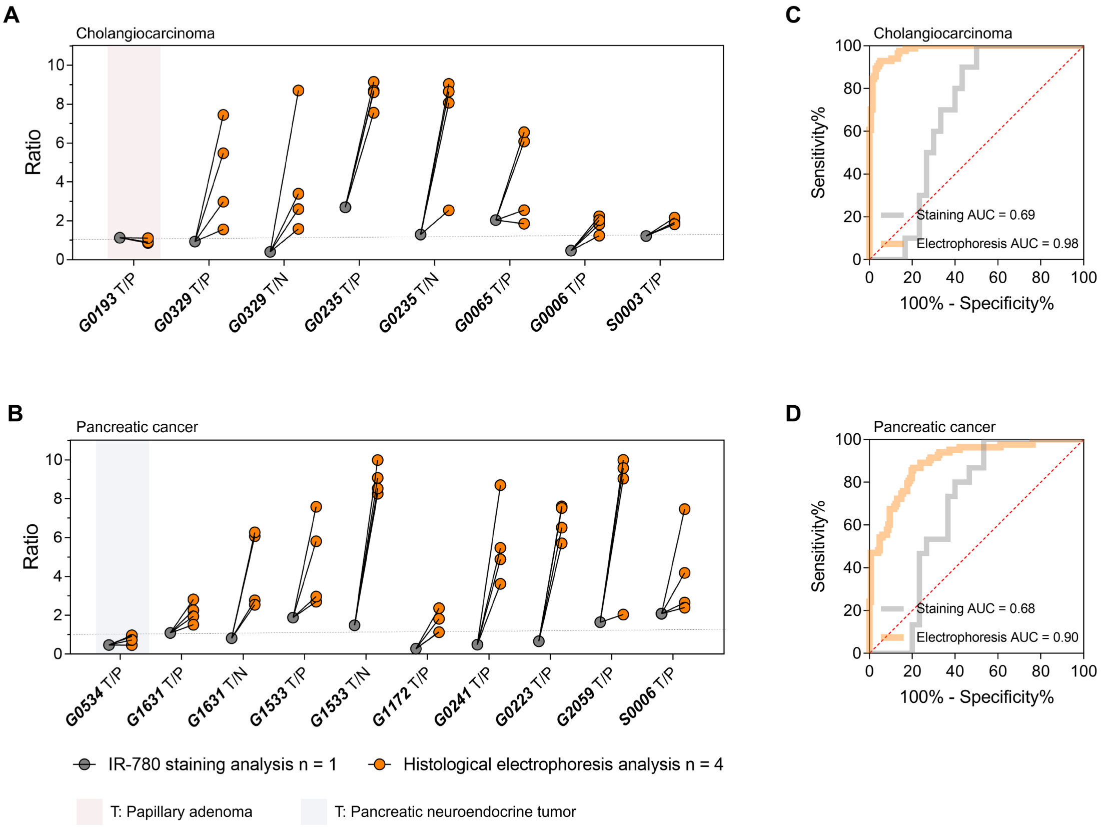

Figure S15. TSD-HE analysis for cholangiocarcinoma and pancreatic cancer. (A and B) Enhancement of tumor-to-paracancerous (or normal) tissue ratios in cholangiocarcinoma and pancreatic cancer via histological electrophoresis analysis. The cholangiocarcinoma specimens are obtained from six patients (**Table S1**, A). The pancreatic cancer specimens are obtained from eight patients (**Table S1**, B). (C) ROC plot of sensitivity% versus false positive rate (100%-specificity) for cancer versus non-cancer classification in data from specimens across above cholangiocarcinoma patients. The AUC is 0.98 for histological electrophoresis analysis versus 0.69 for staining analysis. (D) ROC plot of sensitivity% versus false positive rate (100%-specificity) for cancer versus non-cancer classification in data from specimens across above pancreatic cancer patients. The AUC is 0.90 for histological electrophoresis analysis versus 0.68 for staining analysis.

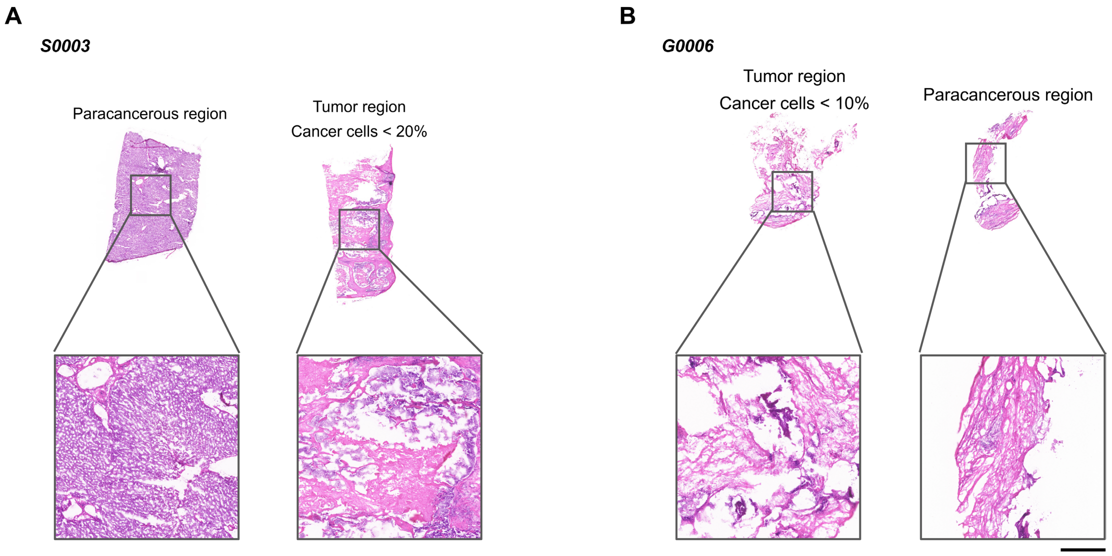

Figure S16. H&E staining results of S0003 and G0006. (A and B) The H&E staining results of S0003 (A) and G0006 (B) are used to confirm the histological type of the tumor, paracancerous, and normal tissue regions within tissue sections. Scale bar: 500 µm.

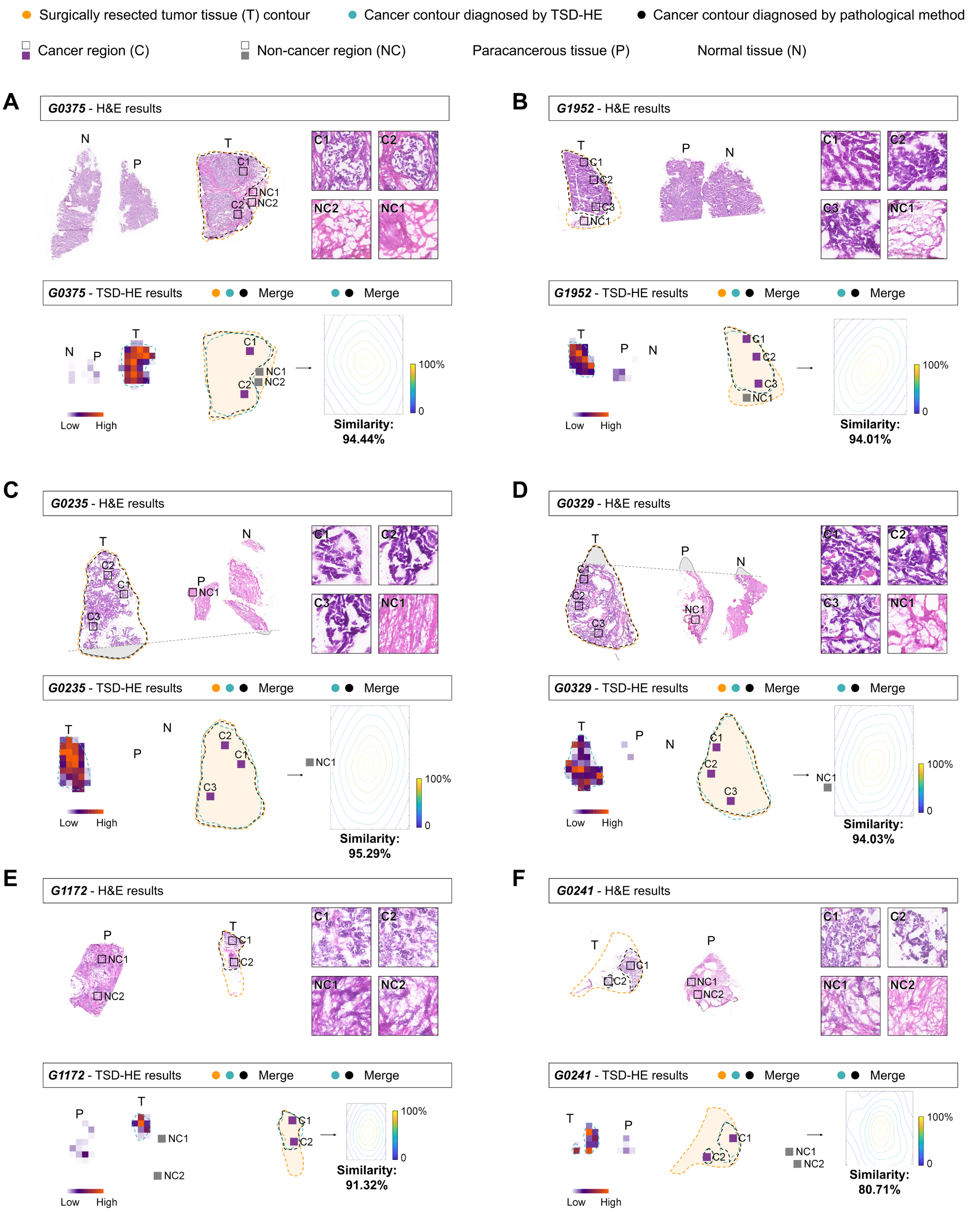

Figure S17. A side-by-side comparison of the TSD-HE strategy and clinical pathological approach. Top of (A-F): H&E staining of the tumor and the corresponding paracancerous and normal tissues with the pathologically confirmed malignant contours and the surgically resected tumor tissue contours. Bottom of (A-F): Positive signal patterns plotted from the signal heat maps post-electrophoresis could accurately diagnose the malignant contours by the TSD-HE system. The merged patterns of the surgically resected tumor tissue contours, the pathologically confirmed malignant contours, the system-diagnosed malignant contours, and the calculated similarity of the pathologically confirmed malignant contours and the system-diagnosed malignant contours. **Data Note:** Here we focus on comparison of

the similarity of the contours reported by TSD-HE and the clinical approach. All diagnostic reported results for the two strategies have been mentioned above.

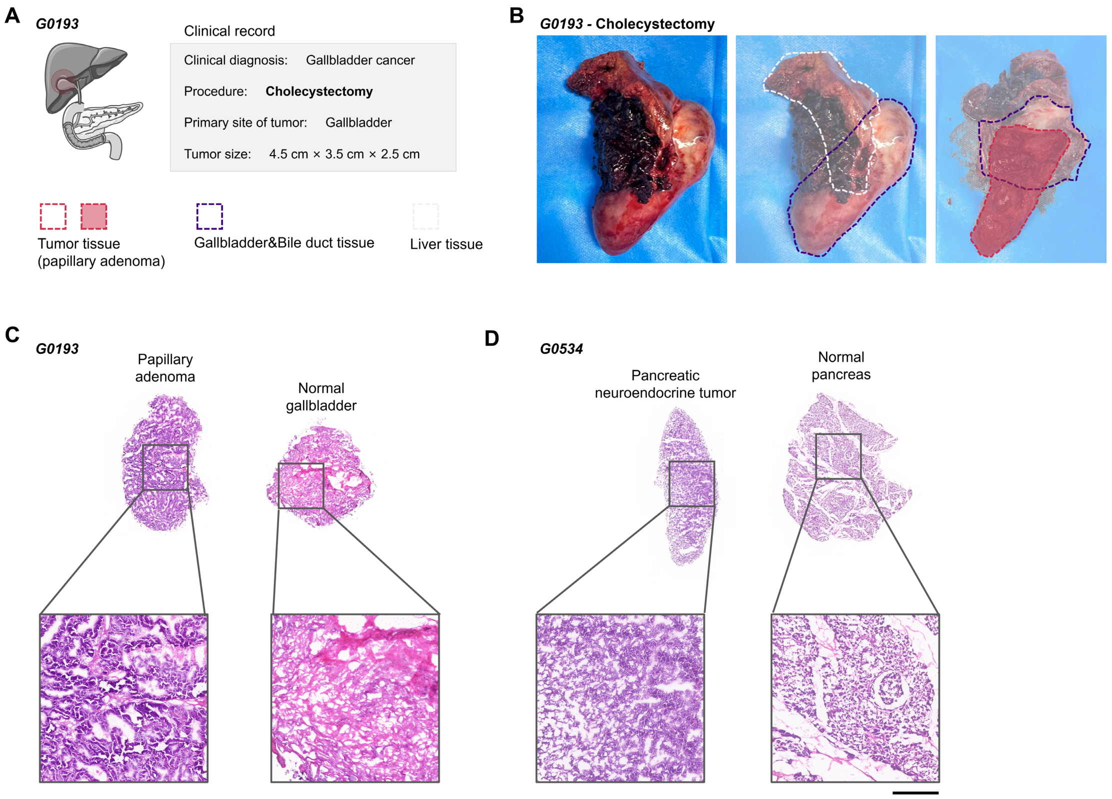

Figure S18. Histological electrophoresis allows us to distinguish the imaging confused benign cyst and malignant tumor. (A) Patient G0193 was pre-surgically imaged and diagnosed as gallbladder cancer and underwent cholecystectomy. (B and C) Photographs (B) and H&E staining results (C) of flesh-resected gallbladder cancer specimens are obtained from G0193. Dashed lines indicate the clinically identified tissue boundary. (C) Tumor region in the analyzed tissue sections of G0193 was histologically confirmed as a papillary adenoma (> 90%, benign cyst). (D) The tumor region in the analyzed tissue sections of G0534 was histologically confirmed as a pancreatic neuroendocrine tumor. Scale bar: 500 µm.
